# Hippocampal granule cell dispersion: a non-specific finding in pediatric patients with no history of seizures

**DOI:** 10.1186/s40478-020-00928-3

**Published:** 2020-04-21

**Authors:** Achira Roy, Kathleen J. Millen, Raj P. Kapur

**Affiliations:** 1 Center for Integrative Brain Research, Seattle Children’s Research Institute, Seattle, Washington USA; 2 Division of Genetic Medicine, Department of Pediatrics, University of Washington, Seattle, Washington USA; 3 Department of Pathology, Seattle Children’s Hospital, Seattle, Washington USA; 4 Division of Pathology, University of Washington, Seattle, Washington USA

**Keywords:** Hippocampus, Granule cell dispersion, Human, Epilepsy, Dentate gyrus, Temporal lobe epilepsy, Gliosis

## Abstract

Chronic epilepsy has been associated with hippocampal abnormalities like neuronal loss, gliosis and granule cell dispersion. The granule cell layer of a normal human hippocampal dentate gyrus is traditionally regarded as a compact neuron-dense layer. Histopathological studies of surgically resected or autopsied hippocampal samples primarily from temporal lobe epilepsy patients, as well as animal models of epilepsy, describe variable patterns of granule cell dispersion including focal cell clusters, broader thick segments, and bilamination or “tram-tracking”. Although most studies have implicated granule cell dispersion as a specific feature of chronic epilepsy, very few “non-seizure” controls were included in these published investigations. Our retrospective survey of 147 cadaveric pediatric human hippocampi identified identical morphological spectra of granule cell dispersion in both normal and seizure-affected brains. Moreover, sections across the entire antero-posterior axis of a control cadaveric hippocampus revealed repetitive occurrence of different morphologies of the granule cell layer – compact, focally disaggregated and bilaminar. The results indicate that granule cell dispersion is within the spectrum of normal variation and not unique to patients with epilepsy. We speculate that sampling bias has been responsible for an erroneous dogma, which we hope to rectify with this investigation.

## Introduction

Chronic epilepsy is often associated with several pathological hippocampal abnormalities [44]. These include sclerosis, characterized by neuronal loss in both dentate gyrus and Ammon’s horn, gliosis, mossy fiber sprouting; as well as granule cell dispersion (GCD), which is observed in the presence or absence of sclerosis [9, 10, 15, 30, 55, 56]. These studies were mostly performed in surgical resections or autopsied brain samples obtained from patients suffering from febrile seizures and temporal lobe epilepsy (TLE); it is unclear whether the putative hippocampal abnormalities are the cause or effect of epilepsy [14, 25].

GCD has been reported commonly in the dentate gyrus (DG) of seizure patients and considered a hallmark of chronic epilepsy, especially TLE [3, 21, 30, 35, 55, 56]. GCD was also reported to be pronounced in sudden unexplained deaths in infants [26, 31]. Many studies using animal models have similarly concluded that hippocampal GCD is a specific feature of chronic epilepsy [40, 43, 60]. The granule cell (GC) layer is generally observed as a highly compact layer of neuronal cell bodies in the dentate gyrus. GCD refers to broadening with or without bilamination of the GC layer, often blurring the boundary between the GC layer and molecular layer of the dentate gyrus [8, 17]. Additionally, some surgical cases also demonstrated the existence of focal clusters of granule cells outside the regular GC layer [54]. GCD has been most consistently correlated with a history of severe febrile or infection-induced seizures during early life (< 4 years age), as well as with duration and frequency of epileptic events [30, 36, 54].

Criteria for determining dispersion vary and depend, in part, on the mode of measurement [8]. A variety of diagnostic criteria for GCD has been suggested, varying from complex morphometric analyses [24, 37] to subjective assessment of dentate gyrus histology [1, 8, 9, 35, 36]. Some reports have suggested abnormal neuronal migration, loss of hilar cells and genetic defects to be responsible for GCD, all influenced by seizure occurrence [10, 30, 54]. However, a substantial cohort of controls, with no history of seizures, was seldom included in the clinical studies; many lacked even 1 control specimen.

Although most reports claim GCD to be a unique feature of epileptic brains, occasional contradictions in either the identification or interpretation of GCD have been published. The first such report identified GCD in two neurologically normal (non-seizure) patients [25]. They suggested that the pathological changes in the DG are not exclusively related to epilepsy, and speculated that GCD may be a separate developmental disorder, independent of TLE [8, 25]. Another study suggested that GCD is not a common result of recurring seizure occurrences originating at an early age, but is more dependent on the type of epileptic syndrome [37]. Finally, there are conflicting data on whether GCD in epileptic patients is due to ectopic GC neurogenesis [6, 20]. At present, there is no consensus over the clinical relevance of GCD or its role in surgical prognosis [8, 9].

We began to question the association between seizures and GCD in the hippocampus, after the phenomenon was observed in cadaveric hippocampi of non-seizure patients. In order to better understand GCD and its clinical correlates, we conducted a retrospective assessment of 147 cadaveric pediatric hippocampi (21 epileptic cases, 126 controls with no history of seizure), with a focus exclusively on the DG. We based our study on the hypothesis that GCD is not specifically associated with epilepsy and may be a variant of normal or a non-specific alteration associated with a wide variety of brain insults.

## Materials and methods

### Human hippocampal samples and clinical records

After appropriate approval from the Institutional Review Board, 147 archived H&E-stained, coronal sections of hippocampus and associated clinical history were obtained from pediatric patients autopsied at Seattle Children’s Hospital between (2014–2019). 126 controls (C, no history of seizures) and 21 seizure (SZ) cases were included in this retrospective study. The following patient-related criteria were abstracted from the archived records: age, gender, post-mortem interval (i.e. duration between time of death and that of autopsy), presence or absence of seizure history (and seizure interval from onset until death for epileptic patients) and major clinical or pathological diagnoses. Corrected age was calculated considering 40 gestational weeks (GW) as day 0. Among the 147 autopsy cases studied, 80 were males and 67 were females, with the corrected age range spanning from 19GW (i.e. −21 weeks) to 20.4 years (i.e. +1060 weeks). Post-mortem interval (PMI) ranged from 2 to 336 h.

### Histological assessment of GCD

Presence or absence of GCD (disaggregated, tram-track) was determined by analyzing hematoxylin-and-eosin (H&E)-stained coronal human hippocampal sections. Archived coronal sections of hippocampus, which were originally prepared as part of routine autopsy examination, were screened retrospectively by a single observer (AR). All available sections (298 sections from 147 patients) were reviewed. For most of the patients, this consisted of a single section (Control: 79/126, 62.70%; Seizure: 12/21, 57.14%); two sections were available for 26 controls (20.63%) and 2 seizure cases (9.52%), and three or more sections for the remainder (Control: 21/126, 16.67%; Seizure: 7/21, 33.33%). Criteria for diagnosis of GCD were the following, irrespective of whether the interfaces between the DG and the hilus or molecular layer were sharp versus diffuse:

- “Disaggregated” GCD: focal broadening of the GC layer (> 120 μm thickness) without any gaps,
- “Tram-track” GCD: focal bilamination of the GC layer with a distinct cell-sparse zone between the inner and outer layers.

In our study, we encountered a few cases with small clusters of “ectopic” granule cells in the hilar region (2 seizure cases, 2 controls) or in the DG molecular layer (1 control, 1 seizure case). These ectopic clusters were always associated with TT or DA.

Any cases deemed initially as definitive or equivocal GCD were re-examined by two observers (AR and RPK) to arrive at a consensus diagnosis. No hippocampal section was excluded from our analysis. Presence versus absence of GCD, along with GCD subtyping, was based on pooled data obtained from all hippocampal sections of a single patient, including sections from both hippocampi when available. For one control (C-20), the entire hippocampus was divided coronally into serial tissue slabs (each ~ 5 mm thick) along the antero-posterior axis. Hematoxylin-eosin (H&E)-stained sections from each slab were examined.

#### Immunohistochemistry

Immunohistochemistry and immunofluorescence were performed on ~ 10 control brains and 6 epileptic brains, chosen based on preliminary tissue histology and sufficient tissue availability. The chosen cases represent a wide range of overlapping ages in the patient and control cohorts (Supplementary Table 1, Additional File 1). Paraffin-embedded, formalin-fixed, hippocampal tissue blocks (~ 4-5 mm thick) were obtained and sectioned at average thickness of 5 μm. Sections were then deparaffinized, as mentioned in the next section, and immunolabelled with mouse anti-CD163 (Leica, Germany; RRID:AB_2756375), rabbit anti-SOX2 (Invitrogen, USA; RRID:AB_2539862), goat anti-PROX1 (R&D systems, USA; RRID:AB_2170716), mouse anti-human CD68 (Dako, Denmark; RRID:AB_2074844) and mouse anti-GFAP (Dako, Denmark; RRID:AB_2109952) using a Ventana Benchmark II automated immunostainer. PROX1 immunolabeling needed extra stringent conditions: treatment with citrate buffer (20 mins, steamer) and overnight antibody incubation at 4 °C. Other antibodies were subjected to mild to standard conditioning and a short 30 mins of antibody incubation at 37 °C. All sections were then treated with biotinylated secondary antibodies (Vector Laboratories, USA, RRIDs: AB_2336123, AB_2313606; Jackson ImmunoResearch Labs, RRID:AB_2338586), and later stained using the DAB method (Vectastain ABC-Peroxidase kits, Vector Laboratories, USA, RRID:AB_2336818). The sections were finally counterstained with hematoxylin.

#### Immunofluorescence

Paraffin-embedded human hippocampal sections were warmed on a hot-plate at approximately 45 °C for 15–20 min, to melt the paraffin. Slides were then immediately transferred to fresh 100% xylene and processed through an ethanol hydration gradient (100, 90, 70, 50% ethanol solutions for 5 min each), before immersion in distilled water. After deparaffinization, immunofluorescence was performed as previously described [48]. Briefly, sections were washed thrice in phosphate buffer saline (PBS), boiled in 10 mM sodium citrate solution for antigen retrieval, blocked in 5% serum in PBS with 0.1% Triton X-100 (0.1% PBX solution) and then incubated overnight at 4 °C with primary antibodies. Sections were then washed thrice in 0.1% PBX solution, incubated with appropriate species-specific secondary antibodies conjugated with Alexa 488, 568 or 647 fluorophores (Invitrogen, USA; RRIDs: AB_2534088, AB_10563566, AB_141778) for 2 h at room temperature and then counterstained with DAPI (4′,6-Diamidino-2-Phenylindole, Dihydrochloride; Invitrogen, RRID:AB_2629482) to visualize nuclei. Sections were cover-slipped using Fluorogel mounting medium (Electron Microscopy Sciences, USA, Cat# 50–247-04). Primary antibodies used: rabbit anti-Calbindin (Swant, Switzerland, RRID:AB_2721225), rat anti-CTIP2 (Abcam, UK, RRID:AB_2064130), rabbit anti-PAX6 (Biolegend, USA, RRID:AB_2565003), rat anti-TBR2 (anti-EOMES, eBioscience, USA, RRID:AB_11042577), rabbit anti-BLBP (Abcam, UK; RRID:AB_2100476), rabbit anti-IBA1 (FUJIFILM Wako Chemicals, USA; RRID:AB_839504). Immunostained sections were imaged in Olympus VS-120 slide-scanner microscope using Olympus VS-Desktop 2.9 software, and later processed in ImageJ 1.51j8 and ImageJ2 (NIH, Bethesda, Maryland, USA) and Olympus VS-Olyvia 2.9 software programs respectively.

Each antibody was validated for immunofluorescence/immunohistochemistry by the correspondent manufacturer, and these data are available publicly on the company websites with indicated catalog numbers. This was also validated by us in our experiments, replicating published/expected expression in appropriate control tissue. No outliers were encountered.

### Quantitative analysis

Data was collected from H&E-stained coronal sections. Thickness and length measurements of human hippocampal GC layer were made using Olympus VS-Olyvia 2.9, Olympus VS-Desktop 2.9 (Olympus Corporation, Tokyo, Japan) and ImageJ2 software programs (NIH, Bethesda, Maryland, USA). Maximum GC layer thickness in each GCD sample was measured at the region where the layer was the thickest. For the ratio measurement of bilaminar GC layers, the entire thickness of the DG was measured – from the edge proximal to the hilus to the edge proximal to the molecular layer. The individual thicknesses of the inner and outer layers were obtained as well. To correlate maximum GC layer thickness versus age, cadaveric sections showing compact, DA and TT phenotypes were compared with corresponding corrected age of death. To plot GCD length as a proportion of total DG length, the inner aspect of the DG was measured for 12 controls and 12 seizure cases, using one coronal hippocampal section with GCD per sample. Statistical significance was assessed using Welch-corrected t-test (for GCD length/Total length plot) and two-way ANOVA followed by Tukey post-hoc test (for GCD proportion plots across groups, age and PMI, GC layer thickness comparison plots, GCD occurrence with clinical diagnoses). Linear regression was used for the maximum GCD thickness versus age of death plots. These analyses were performed in GraphPad Prism v7.0 (GraphPad Software Inc., San Diego, USA) and in Microsoft Excel. Differences were considered significant at *p* < 0.05. Data are represented as stacked and regular bar graphs and scatter plots with mean ± SEM in Fig. 2 and Supplementary Fig. 2 (Additional File 1). Measurements for Supplementary Fig. 6 were obtained with Nikon Elements BR v3–2 (Nikon Corporation, Tokyo, Japan) and final data are represented as mean ± SD.

## Results

### Defining types of GCD in seizure-affected human hippocampi

**Fig. 1:**
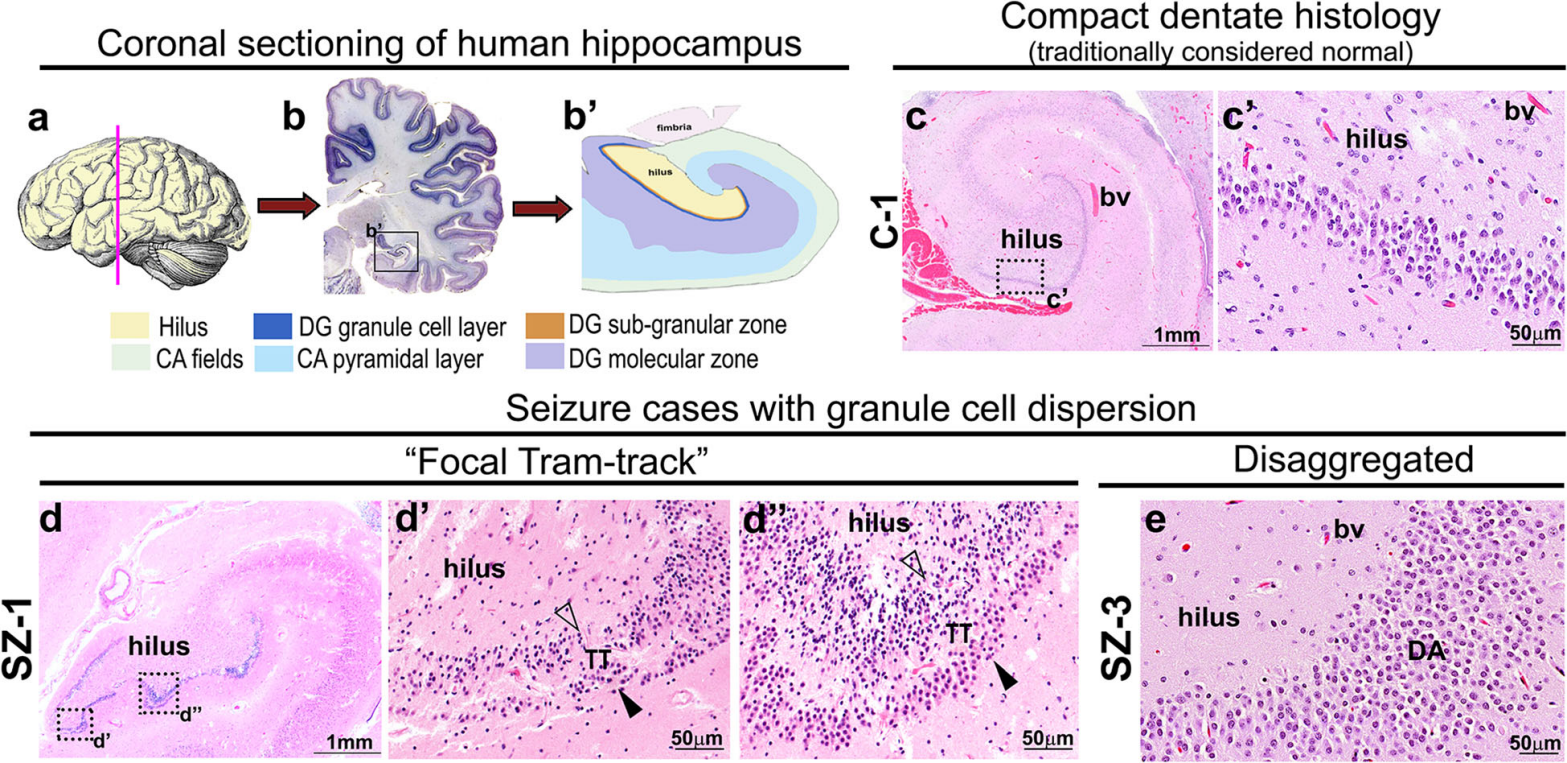
Cadaveric hippocampi reveal a spectrum of GCD in patients with epilepsy. (**a, b, b’**) Schematics explaining the coronal sectioning and structure of human hippocampus. (**c-e**) H&E-stained coronal sections of dentate gyrus (DG) from human cadavers, obtained from SCH archives: representative images demonstrate a typical structure (c) and compactness of the GC layer (c’) in a control human dentate gyrus (with no history of epilepsy/seizure), and GCD in certain seizure cases, ranging from focal “tram-track” (TT) phenotype (d-d”) to more diffused “disaggregated” (DA) form (e). Arrowhead indicates outer granular zone distal to hilus; open arrowhead indicates inner granular zone proximal to hilus; bv, blood vessel. Scalebars: 1 mm (c, d); 50 μm (c’, d’, d”, e)

Stereotypically, the human hippocampal GC layer appears to have a compact, neuron-dense histology, with a sharp boundary separating the molecular layer (Fig. 1b’,c,c’). While studying the cadaveric hippocampal samples from patients with history of epilepsy or seizure, we observed co-existence of compact GC layer and a range of GCD subtypes in the DG (Fig. 1d-d”, e), as originally reported in hippocampal samples of TLE patients [30]. These were later classified by Blumcke et al. into different categories of DG granule cell pathology (GCP) [8]. This classification offered 3 major categories – 1) entire DG appears normal, or non-GCP; 2) Type 1 GCP, characterized by severe cell loss; and 3) Type 2 GCP, that includes at least one DG focus of broadening, clustering or duplication of GC layer [8]. In our study, we further subdivided Type 2 GCP category broadly into two subtypes. The first is marked by broad, less dense GC layer, often with poorly defined borders with the molecular layer; we refer to this as “disaggregated” GCD (DA). The second subtype has a bilaminar appearance of the GC layer with cell-sparse zone in the center [3, 30]; we refer to this as “tram-track” GCD (TT). By studying the H&E-stained hippocampal specimens from patients with seizure history (*n* = 21), we categorized the morphology of GC layer for each as compact (10/21; 47.62%, subtype equivalent to non-GCP of [8]), only DA (5/21; 23.81%), only TT (1/21; 4.76%), or both DA and TT (5/21; 23.81%) (Table 1). Focal granule cell loss was observed in only 1 seizure case (rigorous morphometric analysis of neuronal density was not performed). As previously reported [30], we also encountered dispersion and variation in the pattern and thickness of the GC layer at the angles and enfolded regions of both control and seizure-affected hippocampi. However, these areas were excluded from our consideration of “GCD” and also from our quantitative analyses.

**Table 1:** List of cadaveric epilepsy cases with recorded clinical features. 21 cadaveric epilepsy cases with history of single or multiple events of seizures, accompanied with a table of clinical history obtained from the archives of Seattle Children’s Hospital Department of Pathology (2014–2019), are tabulated. The features analyzed included age of death, gender, post-mortem interval (PMI), presence or absence of GCD subtypes, clinical diagnoses like seizure interval (from seizure onset until death), evidence of malformation/anomaly in the central nervous system (CNS), and/or of other CNS (acquired forms of CNS injury such as hypoxic-ischemic encephalopathy (HIE), cerebral edema) and non-CNS conditions. GW, gestational weeks; TT, tram-track; DA, disaggregated; mo, months

Code	Age of death (GW)	Corrected age of death	Gender	PMI	Seizure (Y/N)	Seizure interval	GCD (Y/N)		Diagnoses		
							TT	DA	CNS malformation/anomaly	Other (CNS)	Non-CNS conditions
**SZ-1**^a^	7 days	7 days	M	12 h	Y	< 24 h	Y	Y	encephalopathy, seizures	–	*Proteus mirabilis* septic shock, coagulopathy and acidosis, hypoxic respiratory failure
**SZ-2**^a^	6 years	6 years	F	10 h	Y	6 years; intractable	N	N	global developmental delay, chronic seizure disorder with static encephalopathy	hypoplastic cerebellum	recurrent pulmonary infections
**SZ-3**^a^	16 years	16 years	F	22 h	Y	13 years	N	Y	severe congenital neuromuscular disorder, complex status epilepticus	severe gliosis and neuronal loss	acute bronchopneumonia, cardiac arrest
**SZ-4**^b^	6 years	6 years	M	not known	Y	> 5 years; multiple episodes till death, intractable	Y	Y	brain overgrowth with polymicrogyria, diffused cortical dysplasia, *AKT3 R465W* mutation, possibly SUDEP	gliosis, mild ventriculomegaly	congenital diaphragmatic hernia, coagulopathy, defects in liver, spleen and kidney
**SZ-5**^a^	3 years	3 years	M	2 h	Y	7 weeks; multiple episodes till death, intractable	Y	N	subclinical status epilepticus	cerebral edema, HIE, anoxic brain injury secondary to pulmonary arrest	pulmonary arrest
**SZ-6**^a^	2 days	2 days	F	21.5 h	Y	2 days (possible seizures)	Y	Y	–	cerebral edema, HIE	acute chorioamnionitis with funisitis and three vessel umbilical vasculitis
**SZ-7**^a^	4 days	4 days	M	38 h	Y	< 24 h	N	Y	–	HIE	severe acidosis, cardiorespiratory failure, visceral anomalies
**SZ-8**^a^	18mo	18mo	M	26 h	Y	1–3 months of age; no further seizures post-treatment of phenobarbital	Y	Y	developmental delay and seizure disorder (2q21.1 duplication)	focal neuronal loss and gliosis in hippocampus	abdominal distension, brady-cardiac arrest, acute pan-lobar pneumonia
**SZ-9**^a^	2 years	2 years	M	72 h	Y	4 months; multiple episodes between first seizure and death	N	N	retinoblastoma with diffuse leptomeningeal spread and direct infiltration of brain and spinal cord	–	pulmonary edema, congestive hepatomegaly
**SZ-10**^a^	5 weeks (28GW)	33GW	F	17.5 h	Y	< 24 h	Y	Y	multiple seizure events	hemorrhage and necrosis secondary to dural venous thrombosis.	necrotizing enterocolitis
**SZ-11**^a^	12 years	12 years	F	96 h	Y	16 months; intractable	N	N	–	cerebral edema, HIE and brain death	appendicitis
**SZ-12**^a^	19 years	19 years	M	216 h	Y	14 years; intractable	N	N	seizure disorder	–	Trisomy 16p/monosomy 9p, respiratory distress syndrome, *Pseudomonas* pneumonia, cardiomegaly
**SZ-13**^a^	7 weeks (32 4/7 GW)	39 4/7 GW	F	14 h	Y	6 weeks (possible seizures)	N	Y	epilepsy (focal apoptotic neurons within DG)	–	severe pulmonary hypertension, veno-occlusive disease
**SZ-14**^a^	8 years	8 years	F	65 h	Y	2 years (sleep EEG done)	N	N	encephalomalacia, hydrocephalus, seizures, developmental delay	–	unbalanced chromosomal translocation, congenital mitral valve stenosis, heart failure
**SZ-15**^a^	7 weeks	7 weeks	F	3 h	Y	6 weeks	N	Y	hypotonia and episodic breathing progressing to seizures	elevated CSF and plasma glycine levels	–
**SZ-16**^a^	17 years	17 years	F	96 h	Y	5 days	N	N	generalized tonic-clonic seizure, brain herniation	acute hemorrhage, edema	Type 1 diabetes, oligoarticular juvenile arthritis, celiac disease
**SZ-17**^a^	17 years	17 years	M	64 h	Y	~ 17 years; intractable	N	Y	spastic quadriplegia, epilepsy, static leukoencephalopathy, ventriculomegaly	white matter gliosis	acute kidney injury, obstructive apnea, hypotonia
**SZ-18**^a^	8 days	8 days	M	42 h	Y	8 days (onset at birth)	N	N	seizures (treated with antiepileptics)	brain herniation, diffuse cerebral edema	ornithine transcarbamylase deficiency, hyperammonemia, hepatosplenomegaly
**SZ-19**^a^	3 years	3 years	F	15.6 h	Y	2.5 years	N	N	severe craniofacial malformations, seizure history	neurological injury, meningitis	–
**SZ-20**^a^	4 years	4 years	M	15.5 h	Y	16 months	N	N	seizures (no recurrence post-treatment with medications), global developmental delay	subacute diffuse CNS hemorrhagic necrosis with massive intraventricular blood clot	multiple chromosomal abnormalities and associated chronic health problems, atypical lymphoid hyperplasia, concerning primary or secondary immunodeficiency
**SZ-21**^a^	9 years	9 years	M	2 h	Y	2 months	N	N	refractory status epilepticus secondary to febrile infection-related status epilepticus, multiple events	diffuse severe gliosis, patchy neuronal loss, dramatic loss of CA1 neurons	–

^a^ based on microscopic evaluation of archived hippocampal sections

^b^ case published in [3, 46]; unused right hemisphere was obtained from SCH morgue and pathological studies were done by RPK on the right hippocampus for the first time for this study

### GCD is evident in brains irrespective of the history of seizures

**Fig. 2:**
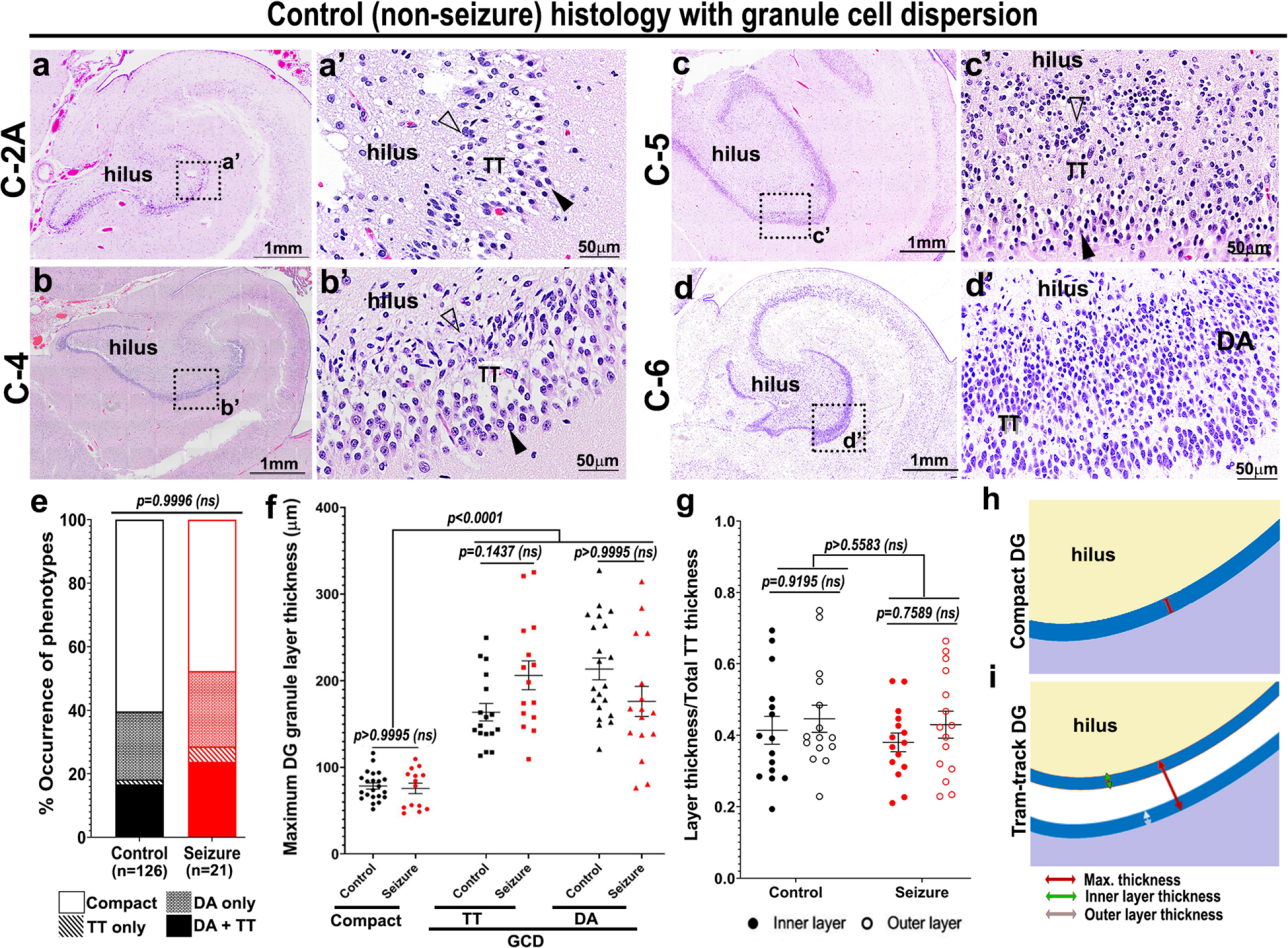
Cadaveric hippocampi reveal the GCD spectrum in controls with no history of seizures. (**a-d’**) H&E staining of coronal hippocampal sections of control human cadavers, with no history of epilepsy or seizure, revealed the entire spectrum of GCD ranging from focal tram-track (TT) to disaggregated (DA) forms, as observed in some seizure cases. Often, TT and DA forms are seen at the same plane of section. Open arrowhead indicates inner granular zone proximal to hilus; arrowhead indicates outer granular zone distal to hilus. (**e**) Frequencies of GCD subtypes in seizure cases (11/21) were not significantly different from that in controls (50/126), as determined by two-way ANOVA, followed by Tukey post-hoc test (p > 0.9996). (**f**) Comparison of the maximum thickness of GC layer between control and seizure hippocampal samples, measured as shown in (**h,i**), revealed no significant differences in all DG subtypes – compact, TT, DA. But the maximum GC thicknesses of both TT and DA sets were significantly higher than the compact ones (p < 0.0001). (**g**) Ratios of inner or outer layer thickness to the total GC thickness, as demonstrated in (**i**), showed no significant difference between control and mutant, as well as within each group. Data are represented as 100% stacked columns (e), mean ± SEM (f) or mean ratio ± SEM (g) in scatter plots; two-way ANOVA followed by Tukey post-hoc test were performed. Differences were considered significant at p < 0.05; ns, not significant. Scalebars: 1 mm (a, b, c, d); 50 μm (a’, b’, c’, d’)

While evaluating age-matched cadaveric controls for seizure-affected brain specimens, we serendipitously identified GCD in some samples. This led us to retrospectively investigate the presence of GCD in a large set of 126 control human brains, with no history of seizures, and compare the findings with those observed in the 21 seizure cases (Additional File 2). We identified both DA and TT subtypes of GCD in control DG, similar to that seen in cadaveric epileptic brains (Fig. 2a-d’, Table 2; Supplementary Fig. 1, Additional File 1). The proportion of each subtype observed in controls was as follows: compact (76/126; 60.32%), only DA (27/126; 21.43%), only TT (2/126; 1.58%), both DA and TT (21/126; 16.67%). Statistical analysis between control and seizure cases, using two-way ANOVA, followed by Tukey post-hoc test, confirmed no significant difference across these 4 groups (*p* > 0.9996; Fig. 2e). Furthermore, we often observed the existence of more than one morphological type of GC layer (compact, DA, TT) in the same hippocampal section, irrespective of the seizure history, consistent with previous reports [8, 21].

**Table 2:** List of cadaveric controls with recorded clinical features, demonstrating GCD. 50 controls with no history of seizures, that demonstrated presence of at least one type of GCD in the studied hippocampal section obtained from the archives of Seattle Children’s Hospital Department of Pathology (2014–2019), are tabulated. Other related clinical features obtained/analyzed were age of death, gender, post-mortem interval (PMI), presence or absence of GCD subtypes, clinical diagnoses like evidence of malformation/anomaly in the central nervous system (CNS), and/or of other CNS (acquired forms of CNS injury such as hypoxic-ischemic encephalopathy (HIE), cerebral edema) and non-CNS conditions. GW, gestational weeks; TT, tram-track; DA, disaggregated; mo, months

Code	Age of death (GW)	Corrected age of death	Gender	PMI	Seizure (Y/N)	GCD (Y/N)		Diagnoses		
						TT	DA	CNS malformation/ anomaly	Other (CNS)	Non-CNS conditions
**C-1**^a^	1 day (37GW)	37GW	F	14 h	N	N	Y (focal DA)	large occipital encephalocele, focal dysplasia	neuronal disorganization, HIE	multiple congenital abnormalities
**C-2A,** **C-2B**^a^	4 weeks (27GW)	31GW	M	32 h	N	Y	Y	–	-	RH-isoimmunization hydrops fetalis, liver failure, respiratory distress
**C-3**^a^	3 weeks (26GW)	29GW	M	23 h	N	Y	Y	–	intraventricular hemorrhage, frontoparietal periventricular leukomalacia	necrotizing enterocolitis and pneumatosis, sepsis
**C-4**^a^	3 weeks	3 weeks	F	57.5 h	N	Y	Y	–	diffuse edema	congenital diaphragmatic hernia, coagulopathy, defects in liver, spleen and kidney
**C-5**^a^	7 weeks (27GW)	28GW	M	12 h	N	Y	Y	–	HIE with periventricular leukomalacia	multi-organ hypoxic ischemic injury
**C-6**^a^	2.5mo (term)	10 weeks	F	12 h	N	Y	Y	–	cerebral atrophy	immunodeficiency disorder of undefined etiology, massive hepatomegaly, active bronchopneumonia, cardiomegaly
**C-7**^a^	15 days (28GW)	30GW	F	6 h	N	Y	Y	–	–	congenital heart disease, acute multifocal pneumonia, congestion and hemorrhage
**C-8**^a^	3mo (35GW)	7 weeks	F	17 h	N	Y	Y	–	cerebral atrophy with HIE, edema	Trisomy 21, severe hepatic fibrosis with cholestasis, pneumonia, cardiac defects, liver and kidney injury
**C-9**^a^	2mo	8 weeks	M	20 h	N	Y	Y	–	remote HIE without acute changes, focal cystic periventricular leukomalacia	neonatal gastroschisis repair, cardiovascular defects
**C-10**^a^	3mo	12 weeks	F	16 h	N	Y	Y	–	subacute HIE with uncal herniation	congenital cardiovascular defects, 15q26-qter deletion, multi-organ hypoxia
**C-11**^a^	7 days (32GW)	33GW	F	15 h	N	Y	Y	–	subdural hematoma, diffuse HIE with widespread gliosis and early mineralization, periventricular leukomalacia, hemorrhage, few pyknotic and karyorrhectic cells noted in hippocampus	cystic necrosis of liver, respiratory failure, congested spleen
**C-12**^a^	2mo (37GW)	5 weeks	F	8 h	N	Y	Y	–	diffuse mild cerebral WM gliosis	complex congenital heart disease, cardiomegaly, aspiration pneumonitis
**C-13**^a^	8 years	8 years	F	7 h	N	N	Y	–	hemorrhagic infarction, mild WM atrophy, DG hypoplasia and neuronal loss, gliosis	methylmalonic acidemia, chronic liver failure, coagulopathy, severe diffuse bronchopneumonia, recurrent fevers
**C-14**^a^	2mo (term)	8 weeks	F	20 h	N	Y	Y	–	remote HIE without acute changes, focal cystic periventricular leukomalacia	neonatal gastroschisis repair, cardiovascular defects, *Clostridium* infection
**C-15**^a^	18 days	18 days	M	13.5 h	N	Y	Y	–	kernicterus involving hippocampi, diffuse gliosis with periventricular eukomalacia	Beckwith-Wiedemann Syndrome, respiratory failure, acute kidney injury, thymic cortical stress
**C-16**^a^	10 weeks (32GW)	2 weeks	M	11 h	N	N	Y	–	–	liver dysfunction of uncertain etiology, cytomegalovirus infection
**C-17**^a^	23 days (25GW)	28GW	M	16.25 h	N	Y	Y	–	severe intracranial hemorrhage	necrotizing enterocolitis, severe pneumonia, pulmonary hemorrhage
**C-18**^a^	6 years	6 years	F	68 h	N	Y	Y	–	craniosynostosis surgery	GLIS3 mutation, hepatic fibrosis
**C-19**^a^	6 years	6 years	M	15 h	N	Y	Y	diffuse infiltrating pontine glioma, mild ventriculomegaly	–	–
**C-20**^b^	12 h (38GW)	38GW	M	38.5 h	N	Y	Y	–	HIE	asystole at birth, bilaterallydilated ureters and bladder, increased extramedullary hematopoiesis
**C-21**^a^	3 days (38GW)	38GW	M	15.75 h	N	N	Y	–	mild HIE and edema	hemorrhagic and necrotic small bowel, anomalies in alimentary tract, liver failure
**C-22**^a^	8 weeks (34GW)	2 weeks	M	8 h	N	Y	Y	mild ventriculomegaly	mild diffuse gliosis of white matter	Pentalogy of Cantrell, left pulmonary artery stenosis
**C-23**^a^	10 years	10 years	M	11 h	N	Y	Y	–		immunodeficiency, *Pseudomonas* and *Aspergillus* infection
**C-24**^a^	3 days (38GW)	38GW	M	15.75 h	N	N	Y	–	mild edema and HIE	hemorrhagic and necrotic small bowel, anomalies in alimentary tract, liver failure
**C-25**^a^	17 years	17 years	M	59 h	N	N	Y	–	–	recurrent B cell lymphoblastic leukemia and aspergillosis
**C-26**^a^	4mo	4mo	M	55 h	N	N	Y	axonal mixed sensory/ motor neuropathy	deafness	growth delay, respiratory distress
**C-27**^a^	2mo (32GW)	term (40GW)	F	58 h	N	N	Y	–	periventricular leukomalacia with acute HIE	cardiopulmonary abnormalities, congenital cardiac anomalies, renomegaly
**C-28**^a^	1 week	1 week	M	45 h	N	N	Y	–	HIE, periventricular leukomalacia with prominent gliosis and neuronal loss	congestion and hemorrhage
**C-29**^a^	10mo	10mo	F	46.5 h	N	N	Y	–	global chronic HIE, hippocampus shows mild loss of neurons in CA1 region	heterotaxy syndrome, complex congenital heart disease
**C-30**^a^	3 years	3 years	M	16.5 h	N	N	Y	–	HIE post cardiac arrest, early necrosis of hippocampus	asthma, acute sepsis, cardiac arrest, stress atrophy
**C-31**^a^	3mo (42GW)	101 days	F	13 h	N	N	Y	–	mild HIE	respiratory distress, pulmonary vein stenosis
**C-32**^a^	14 days (28 week)	30GW	M	16 h	N	N	Y	–	mild HIE, diffuse gliosis in WM	massive subacute hepatic necrosis with iron overload, coagulopathy, chronic neonatal lung disease, multiple organ defects
**C-33**^a^	3 years	3 years	M	20.5 h	N	N	Y	global developmental delay	HIE with edema	myopathy, cardiac failure, respiratory failure, infectious diseases, respiratory distress, sepsis
**C-34**^a^	5weeks (33GW)	39GW	F	84 h	N	N	Y	–	–	necrotizing enterocolitis
**C-35**^a^	18 h	18 h	M	14 h	N	N	Y	–	–	complex congenital heart disease, total anomalous pulmonary venous return, lymphatic distention
**C-36**^a^	17 years	17 years	F	69.5 h	N	Y	Y	medulloblastoma, brain injury related to *Aspergillus* encephalo-meningitis, lateral ventriculomegaly	widespread brain death	pulmonary thrombi and congestion and hepatosplenomegaly
**C-37**^a^	9 days	9 days	F	31 h	N	N	Y	–	HIE, brain injury	liver steatosis
**C-38**^a^	6 h (41 5/7 GW)	1.5 weeks	F	78 h	N	N	Y	–	HIE	cardiac respiratory failure, coagulopathy, anemia, severe metabolic acidosis, in-utero feto-maternal hemorrhage
**C-39**^a^	16mo	16mo	M	15 h	N	N	Y	–	multifocal brain infarction with global HIE (CA1 dispersed)	diffuse adherent bowel, necrotizing soft tissue infections, cardiac arrest history
**C-40**^a^	4 weeks	4 weeks	F	24 h	N	Y	Y	–	mild HIE with mild gliosis	truncus arteriosus
**C-41**^a^	4 days (27GW)	27GW	F	144 h	N	N	Y	–	widespread HIE	splenic congestion
**C-42**^a^	8 years	8 years	F	15 h	N	N	Y	–	subdural hematoma	B-cell acute lymphoblastic leukemia, sepsis, acute kidney injury, cardiac instability
**C-43**^a^	6mo	6mo	M	41.5 h	N	N	Y	–	global remote HIE	Denys-Drash Syndrome, chronic kidney disease, *Pseudomonas* abscess, multiple cardiac arrests
**C-44**^a^	6 years 6mo	6 years 6mo	F	13 h	N	N	Y	diffuse intrinsic pontine glioma	–	–
**C-45**^a^	3 days (40 1/7GW)	40GW	F	40.5 h	N	N	Y	–	–	profound hypoxemic respiratory failure, lung developmental arrest
**C-46**^a^	3 weeks (35GW)	38GW	M	19 h	N	N	Y	–	acute HIE	congenital heart disease, kidney hemorrhage
**C-47**^a^	16 days	16 days	M	10 h	N	N	Y	–	HIE, diffuse WM gliosis, periventricular leukomalacia, subarachnoid hemorrhage	complex congenital heart disease, status post-surgical repair
**C-48**^a^	7 weeks	7 weeks	M	50 h	N	N	Y	–	–	necrotizing enterocolitis
**C-49**^a^	35GW	35GW	M	63 h	N	Y	N	–	–	congenital pulmonary dysplasia, interstitial chromosomal deletion ch17
**C-50**^a^	6 days	6 days	M	69 h	N	Y	N	–	subicular necrosis, acute HIE	22q11.2 chromosomal deletion, DiGeorge syndrome

^a^ based on microscopic evaluation of archived hippocampal sections

^b^ step sections as described in text

### Morphometric measurements of GCD are similar in seizure patients and controls

Measuring the maximum thickness of compact, TT and DA sub-zones of GC layer demonstrated that both types of dispersed DG were significantly thicker than compact DG (*p* < 0.0001) in control and seizure-affected brains; however, no significant difference was observed between control and seizure cases in thicknesses of either the compact or dispersed areas (Fig. 2f,h,i). Moreover, for foci of tram-track GCD, the ratios of the inner or outer layer thicknesses to the total (maximum) GC layer thickness were not significantly different for both control and seizure-affected brains (Fig. 2g,i). Cell migration to the GC layer occurs during the first 8 postnatal months, when immature cells gradually disappear from the sub-granular zone near the hilus [49, 50]. We categorized the age of death into bins and calculated the proportion of control and seizure cases per bin that demonstrated GCD. Statistical analyses showed no significant correlation between GCD occurrence and the age of death, in either control or seizure groups (*p* > 0.2; Supplementary Fig. 2a, Additional File 1). The PMI for the entire cohort largely varied between 2 h and 336 h. Comparison of GCD and non-GCD numbers across different PMIs showed no significant correlation between development of GCD and PMI in both control and seizure groups (Supplementary Fig. 2b, Additional File 1). Comparison of maximum GC layer thickness in compact, DA and TT forms showed that both disaggregated and “tram-track” GC layer thicknesses were wider than the “compact” thickness for both control and seizure brains, across ages of the cadaveric samples (Supplementary Fig. 2c, c', Additional File 1). While other groups showed no correlation with the age of death, the “tram-track”-ed seizure cases (Seizure TT) demonstrated a slightly positive correlation of GC layer thickness with increasing age beyond 60 weeks (R^2^ = 0.4517, *p* = 0.0167; Supplementary Fig. 2c, Additional File 1). Table 3 summarizes the overlapping range of clinical parameters (age of death, PMI, gender proportion) between control and seizure groups used in this study.

**Table 3:** Summary of study parameters. Summary table of variables used in this retrospective study to compare between the control (no seizure history) and seizure groups. Variables shown are corrected age of death, post-mortem interval (PMI), gender and seizure interval (from seizure onset until death). Comparison demonstrated broad overlap especially in the range of age of death and PMI between control and seizure sets

Group	Number of cases	Range of corrected age of death	Range of PMI	Gender (% of total)	Range of seizure interval
**Control**	126	-21 to + 1060 weeks	2 to 336 h	Male: 69 (54.76%); Female: 57 (45.24%)	–
**Seizure**	21	-7 to + 990.7 weeks	2 to 216 h	Male: 11 (52.38%); Female: 10 (47.62%)	< 24 h to 17 years

Further, we segregated the patient history into different diagnostic categories and analyzed whether putative GCD occurrence is dependent on any particular clinical diagnoses (Supplementary Fig. 2d, Additional File 1). Two-way ANOVA revealed no statistically significant correlation between the different diagnostic subgroups of patients and the presence of GCD in either control or seizure cases. We observed higher incidence of diagnoses related to the central nervous system for seizure cases than controls; however due to relatively low sample size of the seizure cases, this was also not considered statistically significant. Finally, we calculated the proportion of GC layer affected by GCD per plane of section in controls and epileptic brains and found no significant differences between them (*p* = 0.801; Supplementary Fig. 2e, Additional File 1). Hence, GCD was found to occur at a similar proportion in both control and seizure-affected brains and this occurrence was largely independent of age, gender, PMI or clinical diagnoses.

### Molecular expression of cells is identical in both control and seizure-affected dentate gyri with GCD

**Fig. 3:**
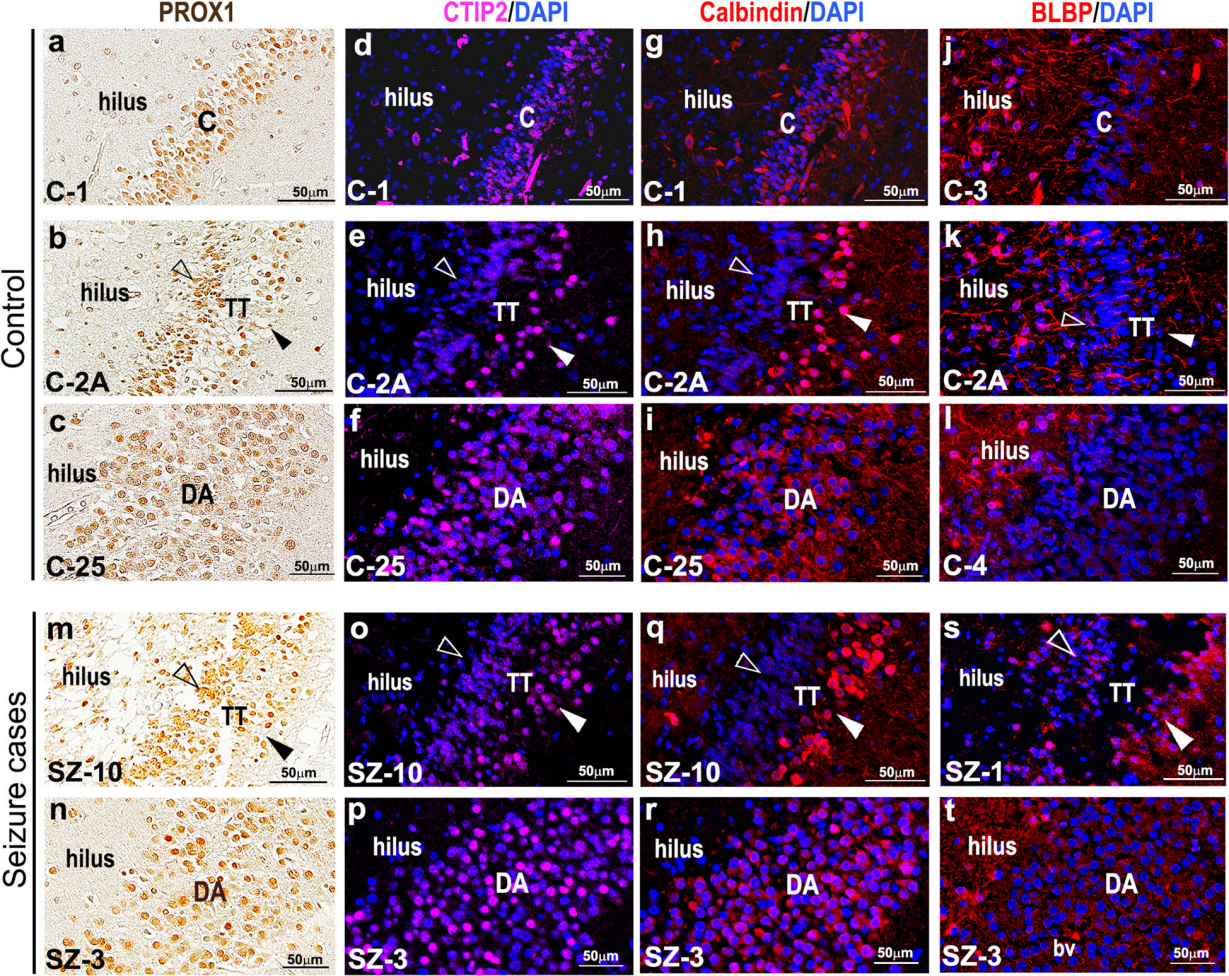
Analysis of cell types in cadaveric control and seizure-affected dentate gyri demonstrating GCD. (**a-t**) Immunohistochemistry studies were performed on coronal paraffin sections of control and seizure-affected cadaveric hippocampi using GC markers, namely PROX1, CTIP2, Calbindin, BLBP. Representative images of compact, tram-track (TT) and disaggregated (DA) DG from both control and seizure cases are demonstrated. No difference was observed between control and seizure brains with respect to molecular expression of PROX1, CTIP2, Calbindin and BLBP across groups. PROX1 was expressed in both inner and outer GC layers of the tram-track DG (**a-c,m,n**). CTIP2 and Calbindin expression is more prominent in the outer layer, compared to the respective inner layers, as seen in compact and tram-track DG (d,e,g,h,o,p); but was expressed in the entire DA zone both in control and seizure cases (**f,i,p,r**). BLBP marked the inner granular layer and the hilar region more densely, as expected (**j-l,s,t**). BLBP staining in SZ-1 showed some non-specific background due to presumed cell autolysis (**s**). TT, tram-track; arrowhead, outer granular zone distal to hilus; open arrowhead, inner granular zone proximal to hilus; bv, blood vessel. Scalebars: 50 μm (**a-t**)

To investigate molecular differences between control and seizure-affected brains, we performed immunohistochemistry and immunofluorescence on the cadaveric hippocampal sections. PROX1, marking cells in the adult GC layer and sub-granular zone [34], was expressed similarly in both control and seizure-affected DG (Fig. 3a-c,m,n). CTIP2 is typically expressed in the hippocampal CA1 and post-mitotic dentate granule cells [47], predominantly in the superficial GC layer; its expression in human DG gets attenuated gradually after mid-gestation [12]. In normal human DG, Calbindin immunostaining is observed in hilus, molecular and GC layers, with strongest expression in the early-born post-mitotic neurons, located in the outer part GC layer distal to the hilar region [2]. In human brains affected by different types of epilepsy and/or hippocampal sclerosis, Calbindin is noted to be prominent in outer granule cells and absent or sparse in the inner layer/half of the GC layer; however no overt cell loss were observed in most of these brains [1]. Similarly in our study, Calbindin and CTIP2 expression was stronger in the outer layer of the compact as well as “tram-track” control and epileptic DG, compared to the inner GC layer (Fig. 3d,e,g,h,o,q). On the other hand, CTIP2 and Calbindin were expressed in the entire “disaggregated” GC layer, in both controls and seizure cases (Fig. 3f,i,p,r). BLBP was expressed in the hilus and the molecular layer of the dentate in both control and seizure-affected brains (Fig. 3j-l, s,t), as expected [38, 53].

To determine whether the inner layer of tram-track DG corresponds to the sub-granular zone harboring progenitors and immature neurons, we examined the distributions of putative neural progenitor markers (SOX2, PAX6, TBR2) in control and seizure-affected hippocampi (Supplementary Fig. 3a-o, Additional File 1) [12, 61]. No overt differences were observed in the expression of these markers within the dispersed (DA or TT) dentate gyri of control or seizure hippocampi. Our data are thus consistent with the study that identified disassociation of proliferation with GCD in the brains of TLE patients [20].

### GCD is not dependent on increased gliosis or injury-driven mechanisms irrespective of seizure occurrence

**Fig. 4:**
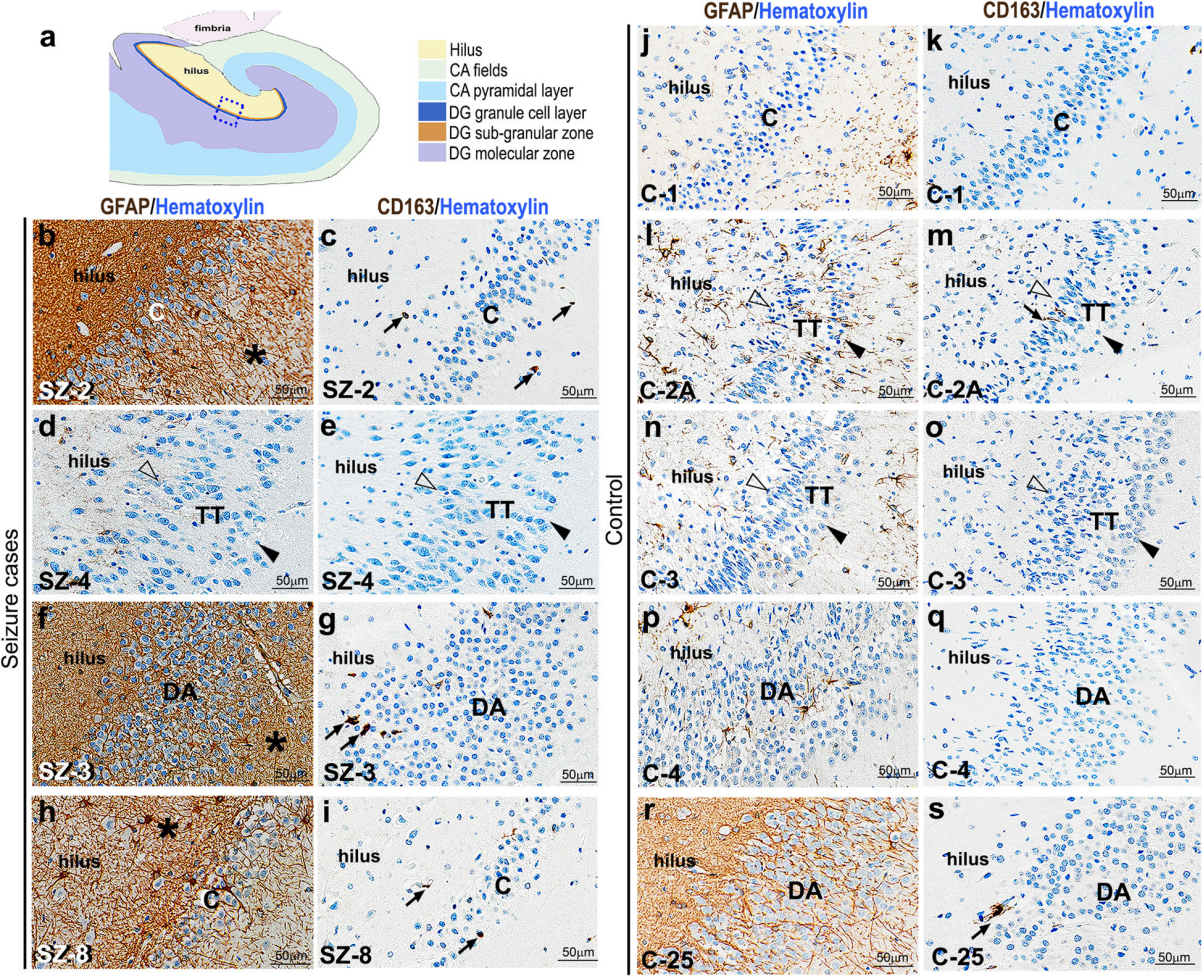
GCD occurrence does not correlate with increased hypoxia/ischemia or gliosis in both control and seizure cases. (**a**) Schematic of coronal human hippocampus; box showed the region of interest represented in (b-o). (**b-s**) Representative images of control and seizure hippocampal samples, with compact (C), tram-track (TT) and disaggregated (DA) DG, studied for injury markers GFAP and CD163. Enhancement of GFAP expression and increase in CD163^+^ cells mark gliosis and M2 macrophages respectively, both indicative of tissue injury. Although there was observable gliosis in most epilepsy brains (asterisk; **b, f, h**), it did not correlate with the occurrence of GCD (**d-g**). SZ-8 demonstrated focal granule cell loss as well as gliosis (**h,i**). Enhanced gliosis was never observed in the studied control sections (j, l, n, p), except mildly in one case (**r**). Although a few CD163^+^ cells were observed in epilepsy and control sections (black arrows; **c, g, i, m, s**), the number of M2 macrophages were not significantly different between control and seizure sections (**c, e, g, i, k, m, o, q, s**). Arrowhead, outer granular zone distal to hilus; open arrowhead, inner granular zone proximal to hilus. Scalebars: 50 μm (**b-s**)

To determine whether tissue injury or macrophage-related inflammation is associated with the GCD phenotypes, we examined the hippocampal expression of injury markers GFAP and CD163 in 6 seizure patients and 10 controls (Fig. 4) [18, 19]. Enhancement of strongly expressing GFAP^+^ and CD163^+^ cells denotes activation of astrocytes (gliosis) and inflammation-related M2 macrophages respectively, both indicative of tissue injury [19, 57]. We observed increased gliosis in some of the cadaveric seizure-affected hippocampi, independent of GCD (Fig. 4b,f,h), although the DG in every epilepsy case did not have increased GFAP^+^ astrocytes (Fig. 4d). Enhanced GFAP expression or gliosis was generally not observed in the control sections, irrespective of presence of GCD (Fig. 4j,l,n,p); among the 10 controls evaluated immunohistochemically, we encountered only 2 control brains that exhibited mild gliosis (as represented in Fig. 4r). We also did not observe any significant differences in the number of CD163^+^ cells in the studied cadaveric control or epileptic hippocampi (Fig. 4c,e,g,i,k,m,o,q,s). Further, we studied the expression of CD68 and IBA1, generic markers for macrophages and microglia respectively, which congregate in response to tissue injury and cellular activation [28, 57]. Similar to CD163, CD68^+^ cells were minimal and expressed non-differentially between control and epileptic brains (Supplementary Fig. 4a-e, k-o; Additional File 1). We did encounter a few IBA^+^ activated microglia, identified by rounded, bushy cellular morphology; but there was no correlation between their presence and GCD or seizure occurrence (Supplementary Fig. 4f-j, p-t; Additional File 1). Thus, our results indicate that occurrence of dispersed GC layer in both controls and seizure cases is not directly associated with either injury or inflammation, although gliosis appeared to be more common in epileptic hippocampi.

### Extent of GCD changes across antero-posterior levels of human hippocampus

**Fig. 5:**
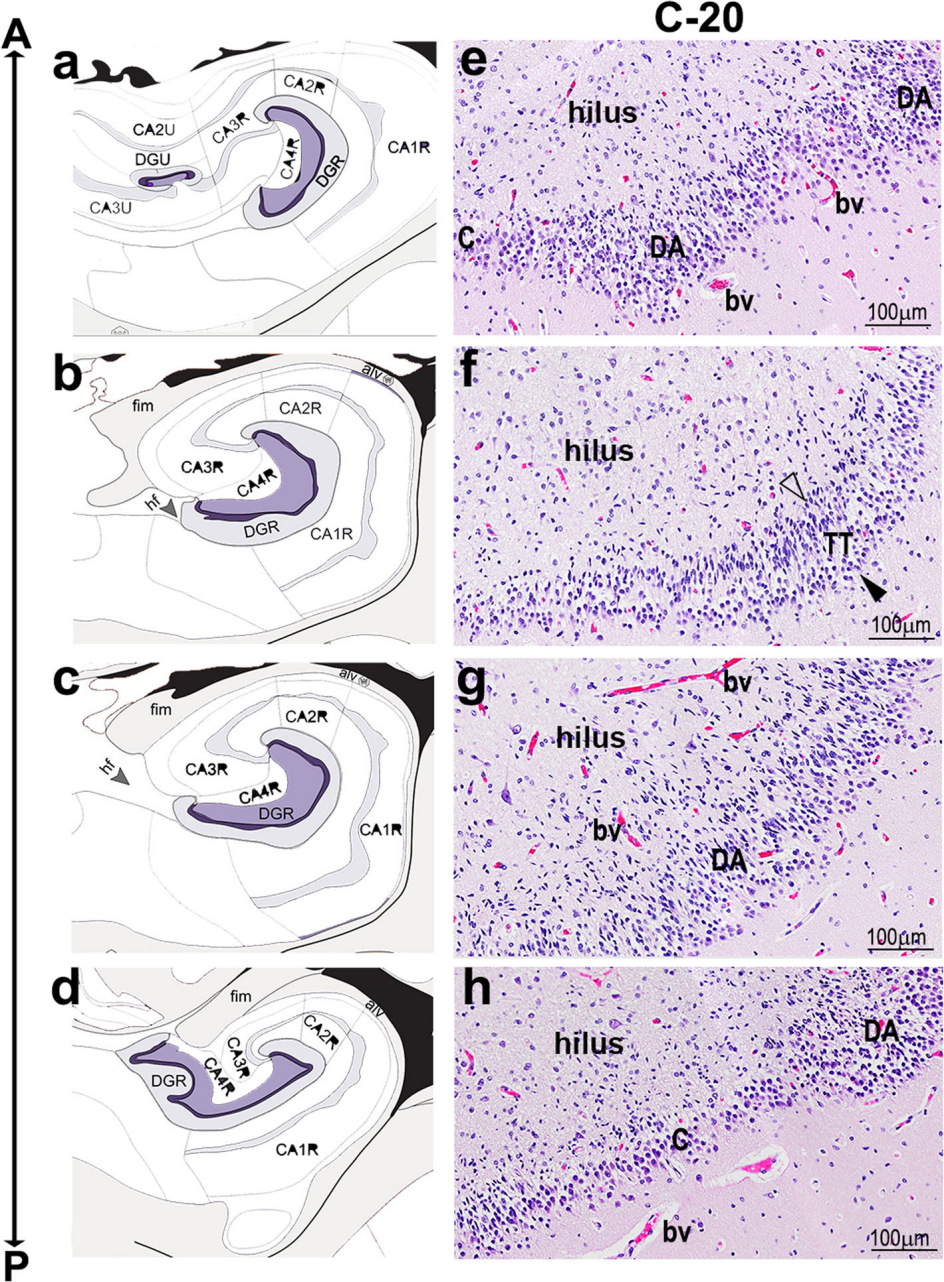
GCD is not consistent across sectioning planes. (**a-d**) Schematics of the human hippocampus, modified from the Allen Human Brain Atlas, at different levels along the antero-posterior (A-P) axis. CA marks subdivisions of cornus ammonis (CA1–4); DG marks the dentate gyrus. The hippocampal morphology changes along the A-P axis. (**e-h**) Representative images of H&E-stained coronal hippocampal sections of C-20, specifically depicting the GC layer. Sectioning plane of each section roughly corresponds to that of the adjacent schematic. The control GC layer demonstrated the entire spectrum of GCD categories: compact (C), disaggregated (DA) and tram-track (TT), often showing co-existence and repetition along the A-P axis. Arrowhead, outer granular zone distal to hilus; open arrowhead, inner granular zone proximal to hilus; bv, blood vessel. Scalebars: 100 μm (e-h)

While analyzing multiple sections from the cadaveric hippocampal paraffin blocks, we observed that GCD was present inconsistently and to variable extents in different sectioning planes of both control and seizure-affected hippocampi (Supplementary Fig. 5a-d", Additional File 1). This suggested that the putative seizure-related nature of “disaggregated” and/or “tram-track” GC layer in the literature might merely be a consequence of sampling bias. As mentioned in the Methods, our designation of controls and seizure cases having or not having GCD was largely based on archived cadaveric histological slides of the hippocampus. To verify the possibility of sampling bias, we coronally sectioned one control hippocampus (C-20) at different levels across the antero-posterior axis, each section a maximum of ~ 5 mm apart from the next. Contours of the hippocampus, including DG, normally change along the antero-posterior axis (Fig. 5a-d). At different points along the same axis we also observed each of the morphological subtypes of the GC layer (Fig. 5e-h). The morphology of GC layer varied from overlapping compact and DA forms to prominent TT, then a combination of TT and DA forms and back to compact forms. Although no formal morphometric analysis was performed, no correlation was suggested between anterior-posterior location or DG contour and any specific pattern of GCD. Instead, GCD appeared to be a sporadic and somewhat randomly distributed variation in DG histology.

Collectively, the data suggest that GCD is a more commonly occurring phenomenon than previously appreciated, and that over-representation of seizure patients in the hippocampal studies may relate to a more thorough sampling in these cohorts. Most importantly, GCD is definitely not an exclusive or characteristic feature of brains affected by epilepsy.

## Discussion

In this retrospective study of 147 cadaveric hippocampi from pediatric patients with seizures and controls without seizure history, we identified certain histopathological characteristics, known to be unique to epileptic brains, in both groups. We found no significant differences in neuronal morphology, molecular expression in different cell types or extent of cell loss between control and seizure-affected hippocampi. Our study challenges the existing dogma in neuropathology that GCD is a specific feature of chronic epilepsy, such as TLE.

### Defining granule cell dispersion (GCD)

The GC layer is a prominent neuronal layer typically composed of a densely packed C-shaped band of cells in a normal hippocampus. A granule cell consists of clusters of apical dendrites, extending through the dentate molecular layer, and basal dendrites, which extend into the hilus and molecular layer in human hippocampus [4, 51]. Granule cell axons (or mossy fibers) project into the hilus to synapse with hilar interneurons, which in turn continue to synapse onto pyramidal neurons in area CA3, thus establishing part of the hippocampal circuitry. The structure and synaptic connectivity of granule cells are vital for brain functions, especially learning and memory consolidation. Ontologically, granule cells localized proximal to the outer molecular layer are born earlier than the ones found at the inner side, adjacent to the hilus [7, 45, 50]. Pathologically, this cell layer is found to be susceptible to hippocampal insults, injury, hypoxia, brain malformations and epilepsy [15, 23, 29]. These conditions often lead to hippocampal sclerosis characterized by neuronal cell loss and reactive gliosis, microglial infiltration and, according to prior studies, GCD [10, 54].

GCD is a histological phenotype reported in the affected dentate gyri of patients suffering from chronic epilepsy [3, 5, 10, 15, 30, 55, 56]. GCD has been described in ~ 40% of the hippocampi of TLE patients [9, 56, 59]. Similar to the human epilepsy-affected brains, animal models of epilepsy demonstrated different kinds of dispersed GC layer, thought to be replicative of human patient pathophysiology [40, 43]. Variable overlapping patterns of GCD have been described. These include focal dispersed clusters of granule cells ectopically present in either hilus or molecular DG layer, segmental broadening and duplication or “tram-tracking” of the GC layer [8, 21, 30]. Some reports categorized bilamination separately from GCD [56]. In our study, both focal broadening and bilamination of GC layer were considered as subtypes of GCD.

Different approaches also exist with regard to the assessment of what should be considered as “GCD”. In our study, we have adapted and modified the GCD classification as proposed by Blumcke et al. [8], where the presence of different grades of GCD was subjectively assessed, primarily based on differential GC density in the DG (focal broadening), blurring of the boundary or ectopic presence of clusters or bilayer of granule cells. Definition of GCD in this manner can be easily translated to routine surgical pathology practice, as it requires no special stains, image analysis or laborious cell counts. Not surprisingly, some variation of the Blumcke et al. (2009) definition has been used in many published studies of GCD and its putative specific association with epilepsy; however, most of these either lacked or included only a few non-seizure controls for comparison (Supplementary Table 2, Additional File 1). Further, we noted that alternative methods of GCD assessment exist. Since dispersed GC layer is generally thicker than “normal”, some studies assessed GCD based on a morphometric method of averaging measurements of GC layer thickness across “straight” parts of DG and then re-averaging values obtained from several sections per hippocampus [24, 37, 52]. In these studies, GCD was defined as an average DG thickness whose value measured greater than a range of average thicknesses obtained from non-seizure controls. As illustrated in Supplementary Fig. 6 (in Additional File 1), this approach, though quantitative, is likely to exclude focal dispersion, which is specifically associated with epilepsy in many publications. Although the morphometric approach may seem less subjective than our study design, determination of what constitutes a “straight” segment of the DG is also highly subjective and may bias against inclusion of focal GCD in these analyses.

### GCD is not exclusively present in seizure-affected brains

Although many reports have accepted the correlative dogma of presence of GCD with seizure history, contradictions exist in the field. One study suggests that GCD is more correlated with learning and memory changes than to seizures, hippocampal sclerosis or neuronal loss [8]. GCD has also been observed in many children, with no history of seizures, who died suddenly and unexpectedly [26, 31, 32]. Although the hippocampal findings in the latter research were interpreted as potentially significant malformations, the possibility of normal variation was not excluded. Another retrospective study of 68 SUDEP (sudden unexpected death in epilepsy) cases showed no evidence of significant differences in hippocampal position or shape or GC abnormalities compared to 53 age-matched non-epilepsy controls, although neither DA nor TT were specifically evaluated [52]. Similarly, a recent study concerning sudden unexplained death in childhood (SUDC) cases also could not clearly relate hippocampal abnormalities to either cause or effect of seizures [39]. We are aware of only one report mentioning the existence of GCD in human patients with no history of epilepsy or seizures [25]. This study reported bilateral GCD in 3 post-mortem pediatric cases, of which only 1 had epilepsy. GCD in the non-seizure brain samples included both DA and TT subtypes, as defined in our study. We also observed GCD in the published images of developing normal human hippocampus, at GW20 and GW 23–25 [12]. However, most human studies, using either surgical resection samples or autopsied hippocampi, lacked substantial number or age-matched controls for a statistically robust comparison.

To develop a comprehensive, comparative study of human hippocampal histopathology, with or without the clinical history of seizures, we performed a retrospective survey of 147 cadaveric cases, with 126 controls (no history of seizure) and 21 cases with clinical history of epilepsy. Our studied cohort is limited to a hospital-based autopsy series of largely pediatric patients, each of whom was diagnosed with one or more comorbidities. We did not have access to “true normal controls”, such as deaths due to sudden trauma or non-hospital based forensic cases. Moreover, being a retrospective study, we did not have control over the postmortem interval to autopsy or brain fixation period, variables that might affect certain molecular analyses. We were also limited to clinical information available in the patients’ medical records. It was impossible to exclude the possibility that some of the controls had undocumented non-convulsive or convulsive seizures or were destined to develop clinical epilepsy had they survived.

In our study, we identified the entire spectrum of GCD in the control dentate gyri, similar in frequency and morphologically indistinct from those observed in the seizure population. This study is the first of its kind to identify GCD in such a large set of human brain samples with no history of seizures. The high prevalence of GCD among the controls in this study and absence of correlation with age, gender, PMI or clinical-pathological diagnoses collectively suggest that GCD, as presently defined, is a variation in normal hippocampal microanatomy and should not be considered as a specific or selective feature of any form of chronic epilepsy, including TLE. Moreover, cell loss was not overtly observed in most of our hippocampal samples, irrespective of GCD, similar to previous report [25]. Thus, our study re-emphasizes the fact that GCD is not a mere consequence of loss of intervening neurons.

### Similar molecular expression patterns in dispersed GC layers of both control and seizure-affected brains raise doubts about putative pathogenesis of GCD

To understand the basis of GCD, a few mechanisms have been proposed using both mouse models and human brain samples affected by epilepsy. In mouse models of febrile and chemical-induced seizures, aberrant migration of either new immature neurons or differentiated mature granule cells were correlated with the occurrence of GCD under the influence of seizures [11, 33, 40]. Defects in GC migration have also been suggested as the basis for GCD in human hippocampi influenced by epileptogenesis [30, 54]. Alternate putative mechanisms include severe cell loss, gliosis/gliogenesis, ectopic sprouting of mossy fibers and abnormal expression of growth factors [15, 35, 37]. Reelin is an important signaling molecule, known to control GC migration by maintaining the radial glial scaffold [22]. Biochemical and functional studies in model systems confirm that formation of a densely packed GC layer is dependent on proper Reelin signaling [58, 62]. Further, human studies as well as genetic and chemical-induced mouse models of epilepsy showed association between reduced *Reelin* mRNA expression and GCD [13, 16, 22, 24, 27, 42]; although there is discrepancy in the type of Reelin^+^ cells (early-born Cajal-Retzius cells or late-born hilar interneurons) considered to be associated with GCD. Introduction of exogenous Reelin into kainate-injected mouse hippocampus was also reported to prevent GCD in mice [41]. However, regardless of whether seizure-related disruption of Reelin signaling, or other factors, influence DG compaction in humans, the morphological consequences of such perturbation with regard to GCD did not appear to be distinguishable from many control hippocampi.

In our retrospective study, we did not observe an overt increase in cell loss or in the number of activated macrophage and microglia (CD163^+^, CD68^+^, IBA1^+^ cells), in either control or seizure-affected hippocampi. Although hippocampi of many epilepsy patients demonstrate DG and/or hilus infiltration by GFAP^+^ astrocytes indicative of injury-driven gliosis, this alteration was absent in 1 of the 4 seizure-affected hippocampi and in all 4 control brain samples with GCD that we tested. Contradictory reports also debate the association of GCD with seizure-induced cell proliferation in the human hippocampus [20, 55]. In our study, no enhancement of neural progenitor markers (SOX2, PAX6, TBR2) was observed in either control or epileptic brains, with or without GCD. Furthermore, we noted prominent expression of Calbindin and CTIP2 in the outer GC layer of both control and seizure cases with “tram-track” GCD. This expression pattern is similar to that seen in normal dentate gyri [1, 12], indicating concentrated presence of mature neurons in the outer GC layer. Such differential expression was however not observed in the “disaggregated” subtype of GCD in control and seizure brains. This suggests that unlike the “tram-track” GCD, the molecular or anatomical distinction between inner and outer GC layers may largely be blurred in the controls and epilepsy brains with “disaggregated” GCD. In summary, these data largely suggest that parameters like cell loss, enhanced proliferation, abnormal neuronal layering, gliosis or microglia/macrophage invasion cannot distinguish GCD between controls and seizure-affected brains, and hence cannot be considered commonly responsible for GCD. Rather, we suspect that GCD represents normal variation in the microanatomic DG structure, which may well be present from or before birth, and have no clinical significance.

### Potential influence of sampling bias on GCD incidence in epilepsy patients

While analyzing multiple sections from the same hippocampal block of control and epilepsy patients for histochemical studies, we encountered intra-hippocampal differences in the extent and severity of the GCD phenotypes. To understand this issue more systematically, we made paraffin blocks of a control cadaveric human hippocampus along the entire antero-posterior length and analyzed multiple representative sections from each block. Intriguingly, we found that the morphology of the hippocampal GC layer varied frequently (within 2-3 mm) and extensively, ranging from compact to disaggregated to bilaminar forms, and also demonstrated combinations of GCD subtypes. The patchy nature of GCD within hippocampi may have contributed to the misconception that GCD is more common in epileptics, particularly those who underwent surgical resection of their hippocampi for TLE. It is very likely that pathologists, and especially researchers, have examined multiple histological sections from the hippocampi of these patients, as opposed to the single hippocampal section evaluated as part of routine autopsy. For example, such variation in the severity of GCD across the antero-posterior axis within a specimen was marked in 23% of the epileptic surgical resection cases studied in [56]. It may be primarily because of the large number of control hippocampi examined in this study with specific attention to DG histology that the fallacy of GCD as a seizure-specific pathological phenotype became evident. We also predict that the percentage of GCD in controls, obtained predominantly by analyzing one autopsy section, may be underrepresented.

## Conclusions

This retrospective human hippocampal survey is the first study to report GCD in a large number of patients without the clinical history of epilepsy. We found no direct association of GCD with seizure or other clinical history, age of death, post-mortem interval or gender. Morphological, histochemical and statistical tests confirmed no overt differences in GCD between control and seizure-affected brains. Most significantly, we determined that the entire morphological spectrum of GC layer exists across the varying hippocampal planes, both in control and epileptic brains. This suggests that the phenomenon of granule cell dispersion is likely a variation of normal hippocampal anatomy that should not be considered a cause or consequence of epilepsy.

## Supplementary information

**Additional file 1.Supplementary Tables and Figures.Supplementary Table 1:** List of patients evaluated with immunohistochemistry/immunofluorescence; **Supplementary Table 2:** List of seizure patients and controls in published reports of GCD; **Supplementary Fig. 1:** Control hippocampi demonstrate variable severities of GCD; **Supplementary Fig. 2:** GCD occurs independent of different clinical parameters in patients with or without epileptic history; **Supplementary Fig. 3:** Distribution of neural progenitors is similar in cadaveric controls and seizure cases with GCD; **Supplementary Fig. 4:** Cadaveric control and seizure brains do not show differential activation of macrophages and microglia; **Supplementary Fig. 5:** GCD severity varies across sectioning planes in both control and seizure brains; **Supplementary Fig. 6:** Alternative assessment method for GCD – averaging across measurements fails to resolve focal forms of GCD.

**Additional file 2.Full list of cases with clinical history.** List of total 147 autopsy cases studied, along with a table of their clinical history obtained from the patient records and histology archives of Seattle Children’s Hospital Department of Pathology (2014-2019). Variables analyzed included age, gender, post-mortem interval, seizure history and interval (onset till death) and major systemic clinical diagnoses including malformations or other problems in central nervous system (CNS), which denotes many acquired forms of CNS injury such as hypoxic-ischemic encephalopathy (HIE), cerebral edema. The blue highlights demarcate the seizure/epilepsy cases tabulated in Table 1. The pink highlights demarcate the controls demonstrating GCD, as tabulated inhippocampal section. GW, gestational weeks; TT, tram-track; DA, disaggregated; PMI, post-mortem interval; hr, hour(s); wk, week(s); mo, month(s); yr, year(s). Table 2. The non-highlighted rows demarcate cadaveric control reports with no clinical history of seizures and no evidence of GCD in any available hippocampal section. GW, gestational weeks; TT, tram-track; DA, disaggregated; PMI, post-mortem interval; hr, hour(s); wk, week(s); mo, month(s); yr, year(s).

## Data Availability

All data generated or analyzed during this study are included in this article and its supplementary information files.

## Abbreviations

C: Compact
C-#: “Control” patient sample
DA: Disaggregated
DG: Dentate gyrus
GC: Granule cell
GCD: Granule cell dispersion
GW: Gestation weeks
h: Hour(s)
PMI: Post-mortem interval
SZ-#: “Seizure” patient sample
TLE: Temporal lobe epilepsy
TT: Tram-track
