# Supplementary material for: Hippocampal granule cell dispersion: a non-specific finding in pediatric patients with no history of seizures
Source: Acta Neuropathol Commun. 2020 Apr 21;8:54. doi: 10.1186/s40478-020-00928-3 (PMC7171777; doi:10.1186/s40478-020-00928-3)
Supplement: Supplementary file 1 — Additional file 1.Supplementary Tables and Figures.Supplementary Table 1: List of patients evaluated with immunohistochemistry/immunofluorescence; Supplementary Table 2: List of seizure patients and controls in published reports of GCD; Supplementary Fig. 1: Control hippocampi demonstrate variable severities of GCD; Supplementary Fig. 2: GCD occurs independent of different clinical parameters in patients with or without epileptic history; Supplementary Fig. 3: Distribution of neural progenitors is similar in cadaveric controls and seizure cases with GCD; Supplementary Fig. 4: Cadaveric control and seizure brains do not show differential activation of macrophages and microglia; Supplementary Fig. 5: GCD severity varies across sectioning planes in both control and seizure brains; Supplementary Fig. 6: Alternative assessment method for GCD – averaging across measurements fails to resolve focal forms of GCD. [file 40478_2020_928_MOESM1_ESM.pdf]

Acta Neuropathologica Communications

Supplementary Material – Additional File 1

Hippocampal granule cell dispersion: a non-specific finding in pediatric patients with no history of seizures

Achira Roy[1], Kathleen J. Millen[1,2], Raj P. Kapur[1,3,4]*

[1]Center for Integrative Brain Research, Seattle Children's Research Institute, Seattle, Washington, USA, email: achira.roy@seattlechildrens.org; [2]Division of Genetic Medicine, Department of Pediatrics, University of Washington, Seattle, Washington, USA, email: kathleen.millen@seattlechildrens.org; [3]Department of Pathology, Seattle Children's Hospital, Seattle, Washington, USA; [4]Division of Pathology, University of Washington, Seattle, Washington, USA, email: raj.kapur@seattlechildrens.org

*Correspondence: Raj P. Kapur, MD, PhD
Department of Laboratories, OC.8.720
Seattle Children's Hospital
4800 Sand Point Way NE
Seattle, WA, USA. ZIP: 98105
Email: raj.kapur@seattlechildrens.org
Tel: +1-206-987-2103

Short Title: Granule cell dispersion in control human dentate gyri

Number of Supplementary Figures: 6

Number of Supplementary Tables: 2

Supplementary Files

Supplementary Table 1: List of patients evaluated with immunohistochemistry/immunofluorescence

This table exclusively includes demographic features of those patients, whose cadaveric hippocampal samples were evaluated with immunohistochemistry/immunofluorescence. Additional information about the clinical history of patients listed in this table is available in Tables 1,2 and Additional File 2. PMI, post-mortem interval; GW, gestational weeks; GCD, granule cell dispersion; mo, month(s).

Supplementary Table 2: List of seizure patients and controls in published reports of GCD

Published reports of GCD were studied; and the number and nature of hippocampal samples from epileptic patients and controls are tabulated along with respective age, if specified. Percentage of epileptic cases or controls demonstrating GCD is also mentioned. This table only includes reports that have at least performed a subjective assessment of the hippocampal pathology. Number of controls specified for each study categorically denotes the ones which do not have any history of seizures. We note that these studies included very few non-seizure controls. Other types of "controls" used have been mentioned in the comment section. PM, post-mortem.

Supplementary Figures:

Supplementary Fig. 1: Control hippocampi demonstrate variable severities of GCD

(**a**) Schematic of a coronal section of human hippocampus. (**b-l'**) H&E-stained images of coronal hippocampal sections of control human cadavers with no history of seizures, demonstrating GCD. The areas within the dotted boxes were magnified (b', c', d', e', f', g', h', i', j', k', l') to demonstrate the presence of GCD, as "tram-track" (TT), disaggregated (DA) or both. Arrowhead, outer granular zone distal to hilus; open arrowhead, inner granular zone proximal to hilus; bv, blood vessel. Scale bars: 1mm (b, c, d, e, f, g, h, i, j, k, l), 50μm (b', c', d', e', f', g', h', i', j', k', l').

Supplementary Fig. 2: GCD occurs independent of different clinical parameters in patients with or without epileptic history

(**a**) Percentage of control hippocampi having GCD (white bars) was not significantly different from that of epileptic hippocampi with GCD (red bars), across corrected age groups ($p>0.2$; age 0 corresponds to term delivery or 40 gestational weeks). Proportion for each group is marked in brackets. (**b**) Statistical analyses across binned post-mortem intervals showed no significant difference in the percentage of GCD and non-GCD groups with respect to PMI, in both controls and seizure cases. (**c, c'**) Maximum thicknesses of control and epileptic "tram-track" (TT), disaggregated (DA) and compact GC layers were plotted across ages of death. No significant correlation of compact and DA thicknesses was observed with age or between control and seizure cases. Seizure TT thickness demonstrated a slightly positive correlation with age of death, beyond 60

weeks (R^2=0.4517, p=0.0167). Compared to compact, DA and TT layer thicknesses were significantly higher for both seizure cases and controls across ages. For better visualization of younger samples, plot of GC layer thickness in patients who died between -20 to +20 weeks of age (rectangle), is magnified in c’. (**d**) Proportion of GCD in controls was similar to that in seizure cases across several clinical diagnoses, as analyzed from patients’ clinical history. (**e**) No significant difference in the proportion of total DG length that developed GCD was observed between control and epileptic hippocampi (p=0.801, t=0.2557, df=18.55). Data are represented as 100% bar graphs (a), 100% stacked columns (b), scatter plots (c, c’), normalized grouped columns (d) or scatter plot with mean ±SEM (e). Statistical analyses performed were two-way ANOVA, followed by Tukey post-hoc test (a, b, d), linear regression fit (c, c’) and Welch-corrected t-test (e). Differences were considered significant at $p<0.05$; ns, non-significant.

Supplementary Fig. 3: Distribution of neural progenitors is similar in cadaveric controls and seizure cases with GCD

SOX2 (**a-c, j, k**), PAX6 (**d-f, l, m**), TBR2 (**g-i, n, o**) are expressed in the nuclei of neural progenitors. Immunohistochemistry of SOX2, PAX6 and TBR2 demonstrated no ectopic presence of progenitors in the GCD-ed dentate gyri of either control or epileptic brains. Blood vessels (bv) also express SOX2, PAX6 and TBR2. Fluorescence observed in the cytoplasm of some brain samples reflects artefacts owing to the fixed cadaveric tissue quality and non-specific antibody staining. TT, tram-track; DA, disaggregated; arrowhead, outer granular zone distal to hilus; open arrowhead, inner granular zone proximal to hilus; white arrow, $TBR2^+$ nucleus. Scalebars: 50μm (a-o).

Supplementary Fig. 4: Cadaveric control and seizure brains do not show differential activation of macrophages and microglia

CD68 (**a-e, k-o**) and IBA1 (**f-j, p-t**) are generic markers of macrophages and microglia respectively, which get activated in response to tissue injury. Although some hippocampal samples exhibited a few IBA1$^+$ activated, rounded or bushy microglia (white arrow; f, g, i, j, p, s, t), no overt differential expression of CD-68 or IBA1 was observed between control and epileptic brains. IBA1 staining in SZ-1 showed some non-specific artefacts due to cell autolysis (h). TT, tram-track; DA, disaggregated; arrowhead, outer granular zone distal to hilus; open arrowhead, inner granular zone proximal to hilus; black arrow, CD68$^+$ cell. Scalebars: 50μm (a-t).

Supplementary Fig. 5: GCD severity varies across sectioning planes in both control and seizure brains

Two H&E-stained coronal sections from different antero-posterior levels of human control (C-11; **a, b**) and epileptic (SZ-1; **c, d**) hippocampi were analyzed. Insets showed the presence of GCD, especially "tram-track" (TT) subtype, in one section (**a', a'', c', c''**) but not in the other (**b', b'', d', d''**), for both control and epileptic brains. DA, disaggregated; C, compact; arrowhead, outer granular zone distal to hilus; open arrowhead, inner granular zone proximal to hilus. Scalebars: 1mm (a, b, c, d); 200μm (a', a'', b', b'', c', c'', d', d'').

Supplementary Fig. 6: Alternative assessment method for GCD – averaging across measurements fails to resolve focal forms of GCD

(**a, b**) Panels depict the same microscopic field of control hippocampus C-20. (a) Focal DA zones are illustrated in the GC layer, as suggested by [4], adopted by many others [1,16] and also applied in our study. (b) A contrasting morphometric approach has been exemplified here, similar to that used by [8,13,15], whereby the average thickness of the dentate GC layer is calculated by taking multiple measurements and GCD is diagnosed only if this value is above the range found in controls. Mean thickness calculated from the measurements in (b) including focal DA regions was statistically not different from that without the DA measurements; the former average had a higher standard deviation as expected. While the latter approach may seem more rigorous, selection of “straight” fields for averaging is subjective, with a tendency of deliberately excluding foci of DA or TT unless they significantly shift the mean thickness.

Supplementary Table 1: List of patients evaluated with immunohistochemistry/immunofluorescence

Code/Group	Age of death (GW)	Corrected age of death	Gender	PMI	Seizure (Y/N)	Seizure onset (interval before death)	GCD (Y/N)
Seizure cases with GCD:							
SZ-1[a]	7days	7days	M	12 h	Y	<24 h	Y
SZ-3[a]	16years	16years	F	22 h	Y	13years	Y
SZ-4[b]	6years	6years	M	not known	Y	>5years; multiple episodes till death, intractable	Y
SZ-8[a]	18mo	18mo	M	26 h	Y	1-3 months of age; no further seizures post-treatment of phenobarbital	Y
SZ-10[a]	5weeks (28GW)	33GW	F	17.5 h	Y	<24 h	Y
Seizure cases without GCD:							
SZ-2[a]	6years	6years	F	10 h	Y	6years; intractable	N
Controls with GCD:							
C-1[a]	1day (37GW)	37GW	F	14 h	N	N	Y (focal DA, mostly compact)
C-2A, C-2B[a]	4weeks (27GW)	31GW	M	32 h	N	N	Y
C-3[a]	3weeks (26GW)	29GW	M	23 h	N	N	Y
C-4[a]	3weeks	3weeks	F	57.5 h	N	N	Y

C-14 [a]	2mo (term)	8weeks	F	20 h	N	N	Y
C-16 [a]	10weeks (32GW)	2weeks	M	11 h	N	N	Y
C-17 [a]	23days (25GW)	28GW	M	16.25 h	N	N	Y
C-18 [a]	6years	6years	F	68 h	N	N	Y
C-19 [a]	6years	6years	M	15 h	N	N	Y
C-25 [a]	17years	17years	M	59 h	N	N	Y

[a] based on microscopic evaluation of archived hippocampal sections

[b] case published in [14,2]; unused right hemisphere was obtained from SCH morgue and pathological studies were done by RPK on the right hippocampus for the first time for this study

Supplementary Table 2: List of seizure patients and controls in published reports of GCD [a]

Study [References]	Seizure Patients			Non-Seizure Controls*			Comment
	Total	Age Range	GCD (%)	Total	Age Range	GCD (%)	
Houser, 1990 [10]	15 (surgical)	18-46 years	11 (73.33%)	6 (PM)	44-77 years	0 (0%)	Both DA and TT subtypes included. Number of sections analyzed were ~20 per brain.
Lurton *et al.*, 1997 [11]	17 (surgical)	11-37 years	8 (47.06%)	4 (PM)	>37 years	0 (0%)	Number of sections studied per patient not specified.
El Bahh *et al.*, 1999 [6]	20 (surgical)	27± 8 years	9 (45%)	2 (surgical)	not specified	0 (0%)	2 tumor resections were taken as controls (age: 48y, 69y). Number of sections studies was not specified.
Harding and Thom, 2001 [9]	1 (PM)	2.5 years	1	2 (PM)	12-20 weeks	2 (100%)	Bilateral GCD observed in an epileptic patient and 2 controls. *Only study that reported GCD in controls.*
Thom *et al.*, 2002 [18]	206 (surgical): 183 (HS), 23 (TL lesion)	15-58 years	~81%	6 (PM)	not specified	0 (0%)	Only DA subtype was considered as "GCD" in this study. However, TT subtype was observed in 10.3% of patients. ~10 consecutive sections with 4mm interval were studied from both seizure and control samples
Thom *et al.*, 2005 [17]	20 (surgical)	17-48 years	14 (70%)	5 (PM)	34-85 years	0 (0%)	-
Blumcke *et al.*, 2007 [5]	178 (surgical)	38.4 ± 13 years	79 (44.38%)	8 (PM)	49 ± 6.8 years	0 (0%)	10% neuronal loss was found within the first standard deviation of age-matched control individuals.
Blumcke *et al.*, 2009 [4]	96 (surgical)	38.2 ± 13.5 years	49 (51.04%)	0	-	-	11 "no-GCP" epilepsy cases were considered as "controls".
Bae *et al.*, 2010 [3]	26 (surgical)	16-43 years	20 (76.92%)	0	-	-	Number of sections studied per patient not specified.

Marucci *et al.*, 2010 [12]	14 (surgical)	19-54 years	7 (50%)	0	-	-	-
Abraham *et al.*, 2011 [1]	28 (surgical)	17-61 years	16 (57.14%)	8 (3 PM, 5 surgical)	not specified	0 (0%)	5 out of 8 "controls" have temporal lobe tumors. Number of immuno-stained sections studied varied from 3 to 9 per patient.
Freiman *et al.*, 2011 [7]	10 (surgical)	31-50 years	4 (40%)	0	-	-	Intact portions ("compact") of TLE GC layer were used as internal controls. Number of sections studied per patient varied between 1 and 6.

[a] List comprises of studies that have used some variation of Blumcke *et al.* (2009) classification of GCD [4], like ours. Reports that used exclusively the approach of averaging GC layer thickness to determine the existence of GCD were excluded from this list.

References

1. Abraham H, Richter Z, Gyimesi C, Horvath Z, Janszky J, Doczi T, Seress L (2011) Degree and pattern of calbindin immunoreactivity in granule cells of the dentate gyrus differ in mesial temporal sclerosis, cortical malformation- and tumor-related epilepsies. Brain Res 1399:66-78. doi:10.1016/j.brainres.2011.05.010
2. Alcantara D, Timms AE, Gripp K, Baker L, Park K, Collins S, Cheng C, Stewart F, Mehta SG, Saggar A, Sztriha L, Zombor M, Caluseriu O, Mesterman R, Van Allen MI, Jacquinet A, Ygberg S, Bernstein JA, Wenger AM, Guturu H, Bejerano G, Gomez-Ospina N, Lehman A, Alfei E, Pantaleoni C, Conti V, Guerrini R, Moog U, Graham JM, Jr., Hevner R, Dobyns WB, O'Driscoll M, Mirzaa GM (2017) Mutations of AKT3 are associated with a wide spectrum of developmental disorders including extreme megalencephaly. Brain 140:2610-2622. doi:10.1093/brain/awx203
3. Bae EK, Jung KH, Chu K, Lee ST, Kim JH, Park KI, Kim M, Chung CK, Lee SK, Roh JK (2010) Neuropathologic and clinical features of human medial temporal lobe epilepsy. Journal of clinical neurology 6:73-80. doi:10.3988/jcn.2010.6.2.73
4. Blumcke I, Kistner I, Clusmann H, Schramm J, Becker AJ, Elger CE, Bien CG, Merschhemke M, Meencke HJ, Lehmann T, Buchfelder M, Weigel D, Buslei R, Stefan H, Pauli E, Hildebrandt M (2009) Towards a clinico-pathological classification of granule cell dispersion in human mesial temporal lobe epilepsies. Acta Neuropathol 117:535-544. doi:10.1007/s00401-009-0512-5
5. Blumcke I, Pauli E, Clusmann H, Schramm J, Becker A, Elger C, Merschhemke M, Meencke HJ, Lehmann T, von Deimling A, Scheiwe C, Zentner J, Volk B, Romstock J, Stefan H, Hildebrandt M (2007) A new clinico-pathological classification system for mesial temporal sclerosis. Acta Neuropathol 113:235-244. doi:10.1007/s00401-006-0187-0
6. El Bahh B, Lespinet V, Lurton D, Coussemacq M, Le Gal La Salle G, Rougier A (1999) Correlations between granule cell dispersion, mossy fiber sprouting, and hippocampal cell loss in temporal lobe epilepsy. Epilepsia 40:1393-1401. doi:10.1111/j.1528-1157.1999.tb02011.x

7. Freiman TM, Eismann-Schweimler J, Frotscher M (2011) Granule cell dispersion in temporal lobe epilepsy is associated with changes in dendritic orientation and spine distribution. Exp Neurol 229:332-338. doi:10.1016/j.expneurol.2011.02.017
8. Haas CA, Dudeck O, Kirsch M, Huszka C, Kann G, Pollak S, Zentner J, Frotscher M (2002) Role for reelin in the development of granule cell dispersion in temporal lobe epilepsy. J Neurosci 22:5797-5802. doi:20026621
9. Harding B, Thom M (2001) Bilateral hippocampal granule cell dispersion: autopsy study of 3 infants. Neuropathology and applied neurobiology 27:245-251
10. Houser CR (1990) Granule cell dispersion in the dentate gyrus of humans with temporal lobe epilepsy. Brain Res 535:195-204
11. Lurton D, Sundstrom L, Brana C, Bloch B, Rougier A (1997) Possible mechanisms inducing granule cell dispersion in humans with temporal lobe epilepsy. Epilepsy Res 26:351-361. doi:10.1016/s0920-1211(96)01002-9
12. Marucci G, Rubboli G, Giulioni M (2010) Role of dentate gyrus alterations in mesial temporal sclerosis. Clinical neuropathology 29:32-35. doi:10.5414/npp29032
13. Mathern GW, Kuhlman PA, Mendoza D, Pretorius JK (1997) Human fascia dentata anatomy and hippocampal neuron densities differ depending on the epileptic syndrome and age at first seizure. Journal of neuropathology and experimental neurology 56:199-212. doi:10.1097/00005072-199702000-00011
14. Riviere JB, Mirzaa GM, O'Roak BJ, Beddaoui M, Alcantara D, Conway RL, St-Onge J, Schwartzentruber JA, Gripp KW, Nikkel SM, Worthylake T, Sullivan CT, Ward TR, Butler HE, Kramer NA, Albrecht B, Armour CM, Armstrong L, Caluseriu O, Cytrynbaum C, Drolet BA, Innes AM, Lauzon JL, Lin AE, Mancini GM, Meschino WS, Reggin JD, Saggar AK, Lerman-Sagie T, Uyanik G, Weksberg R, Zirn B, Beaulieu CL, Majewski J, Bulman DE, O'Driscoll M, Shendure J, Graham JM, Jr., Boycott KM, Dobyns WB (2012) De novo germline and postzygotic mutations in AKT3, PIK3R2 and PIK3CA cause a spectrum of related megalencephaly syndromes. Nat Genet 44:934-940. doi:10.1038/ng.2331
15. Somani A, Zborovschi AB, Liu Y, Patodia S, Michalak Z, Sisodiya SM, Thom M (2019) Hippocampal morphometry in sudden and unexpected death in epilepsy. Neurology 93:e804-e814. doi:10.1212/WNL.0000000000007969
16. Thom M (2014) Review: Hippocampal sclerosis in epilepsy: a neuropathology review. Neuropathology and applied neurobiology 40:520-543. doi:10.1111/nan.12150
17. Thom M, Martinian L, Williams G, Stoeber K, Sisodiya SM (2005) Cell proliferation and granule cell dispersion in human hippocampal sclerosis. Journal of neuropathology and experimental neurology 64:194-201. doi:10.1093/jnen/64.3.194
18. Thom M, Sisodiya SM, Beckett A, Martinian L, Lin WR, Harkness W, Mitchell TN, Craig J, Duncan J, Scaravilli F (2002) Cytoarchitectural abnormalities in hippocampal sclerosis. Journal of neuropathology and experimental neurology 61:510-519. doi:10.1093/jnen/61.6.510

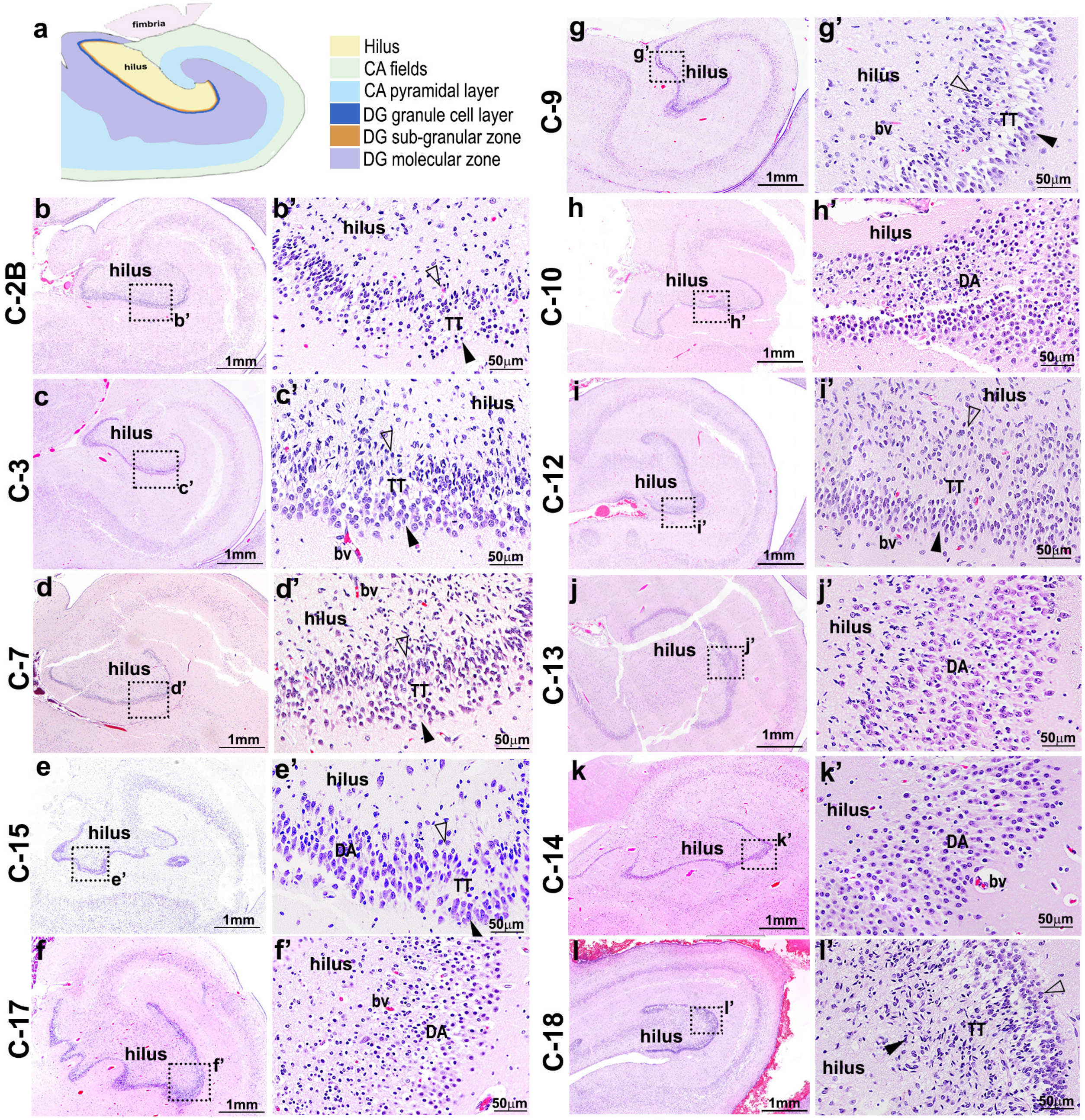

Supplementary Fig. 1

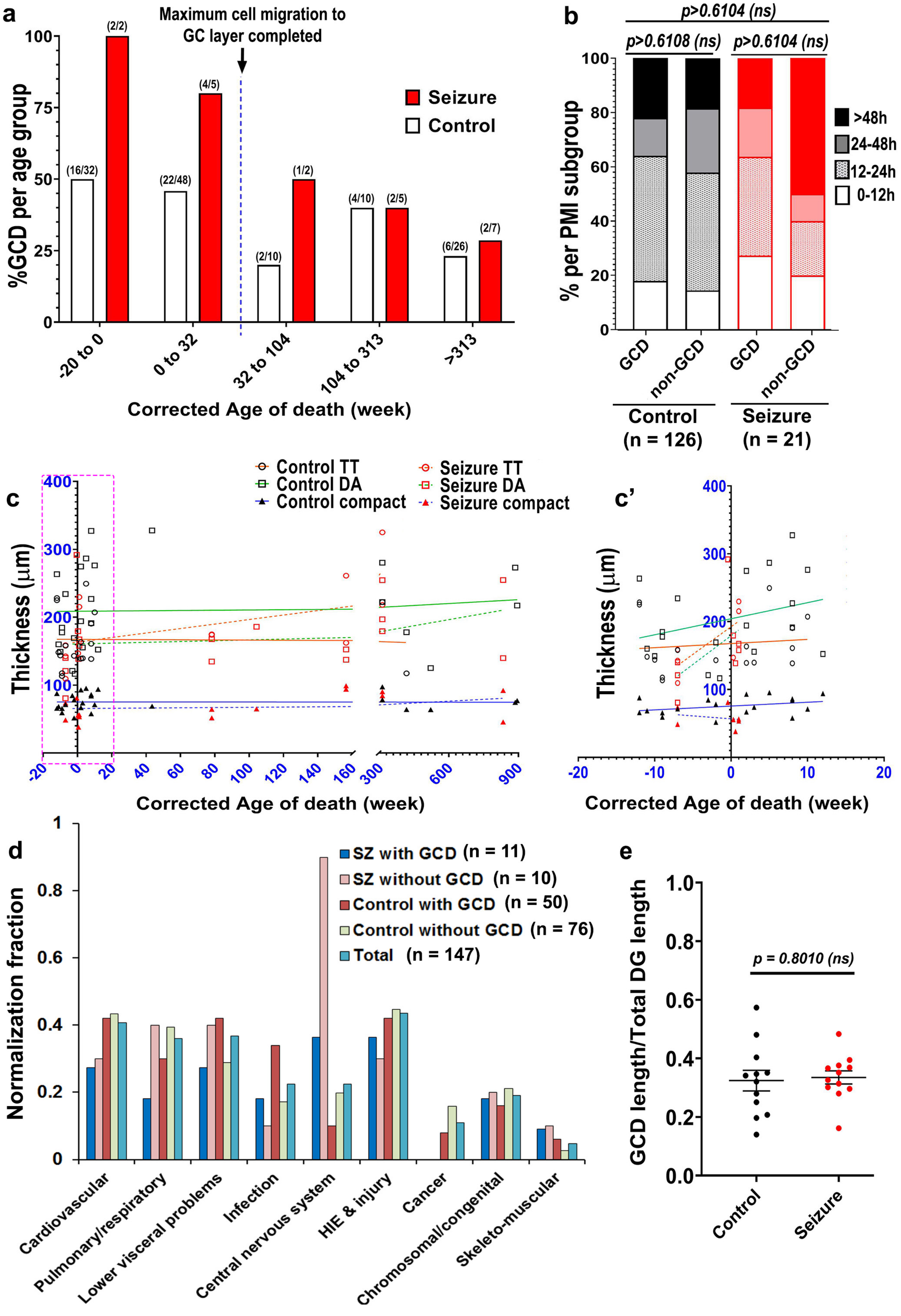

Supplementary Fig. 2

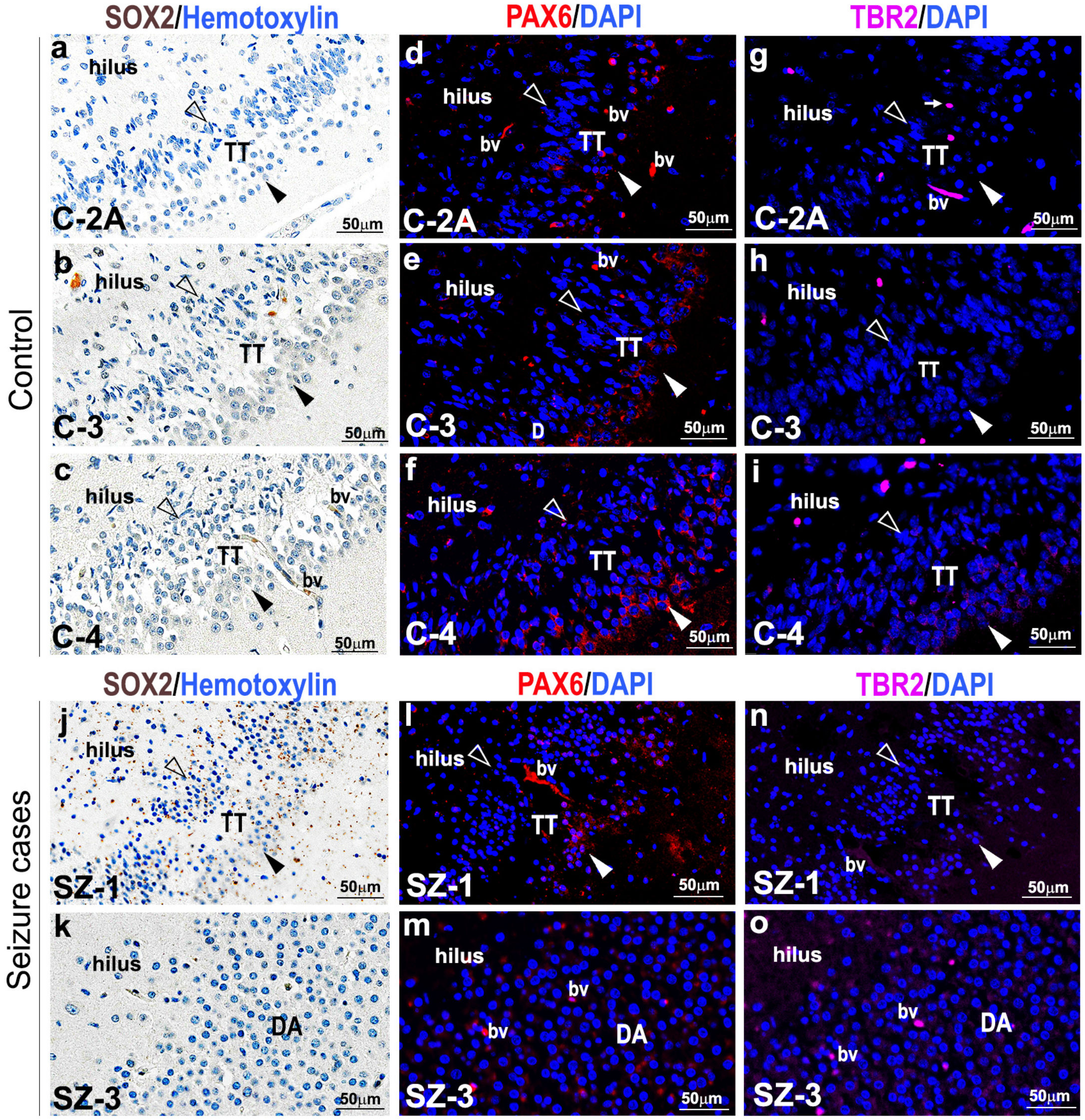

Supplementary Fig. 3

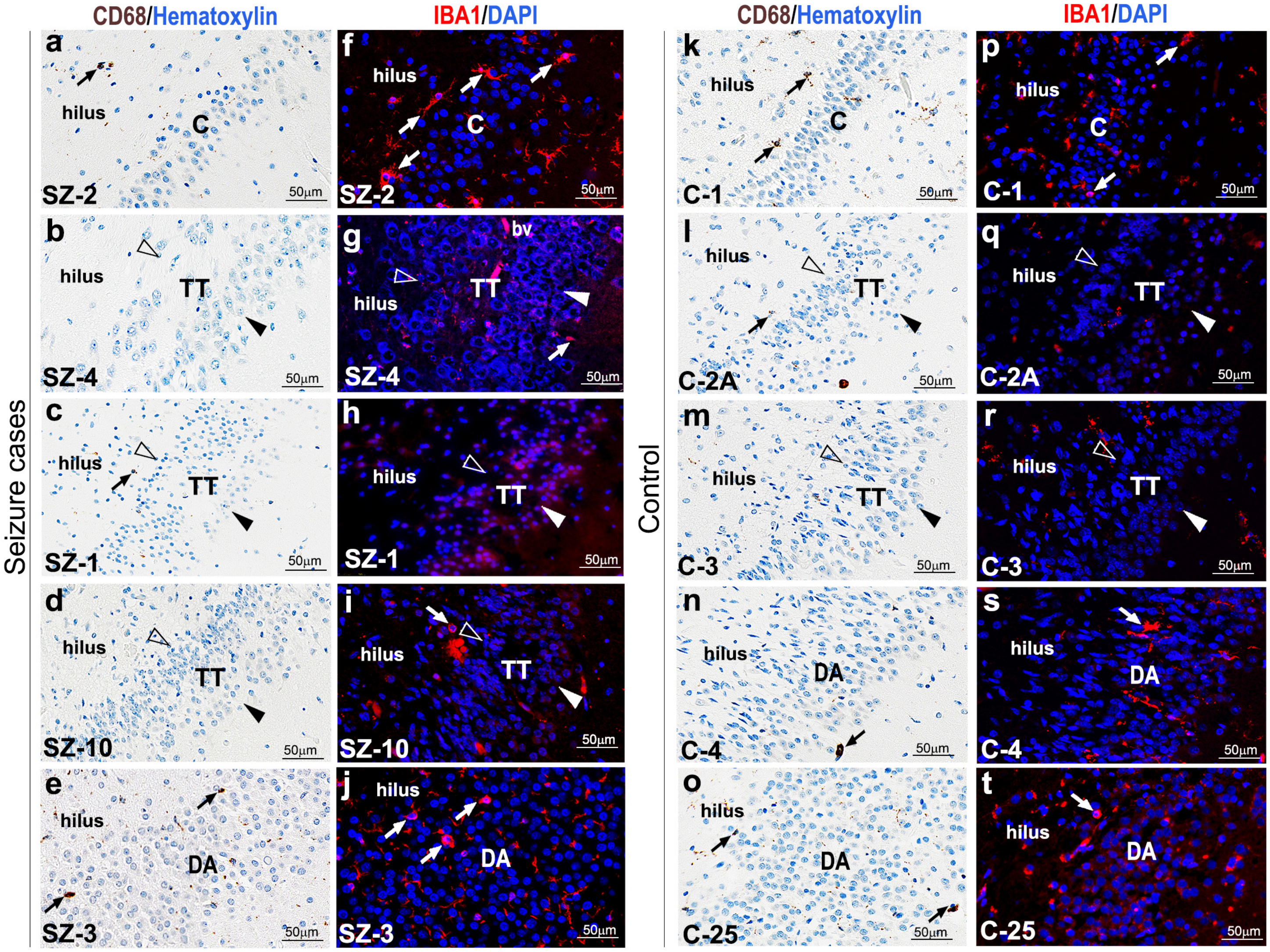

Supplementary Fig. 4

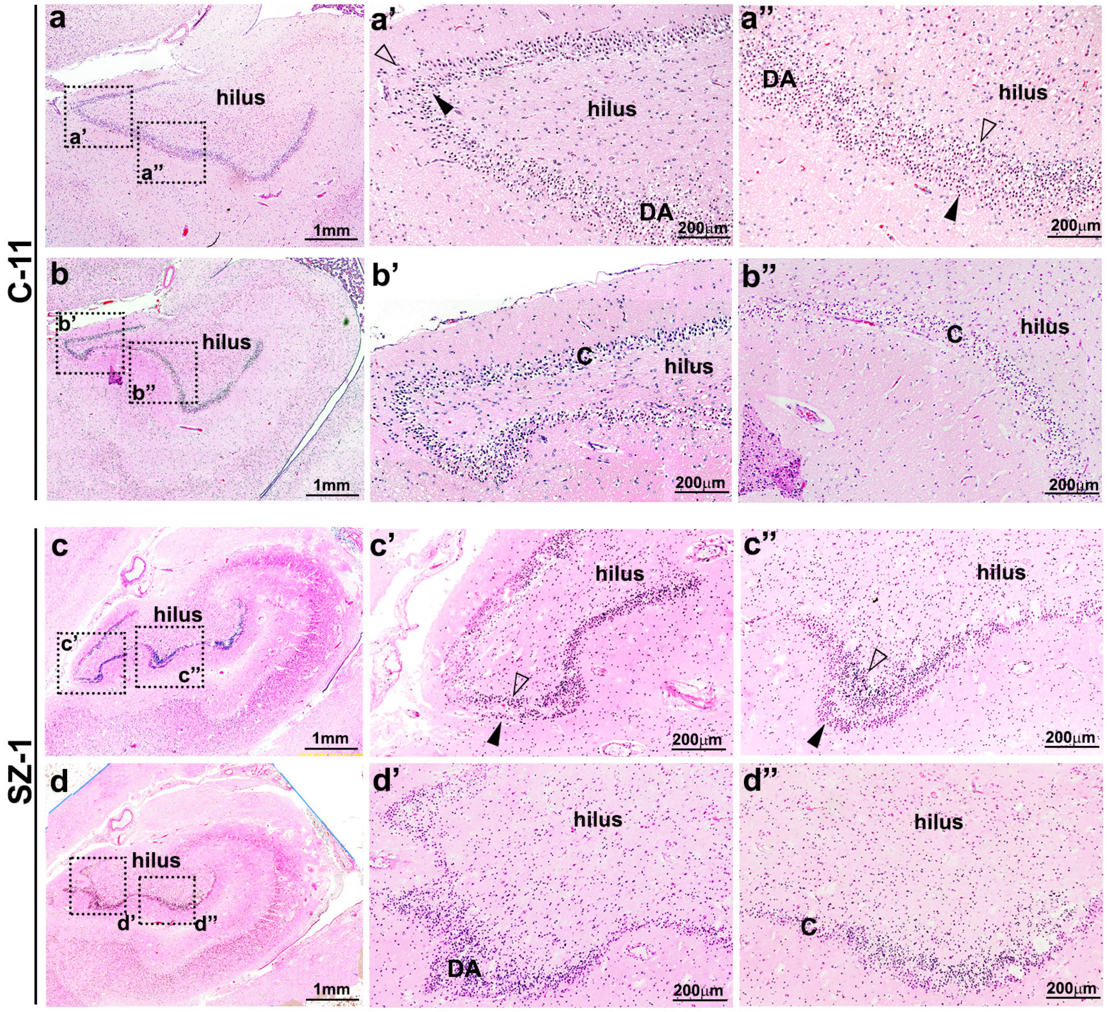

Supplementary Fig. 5

C-20

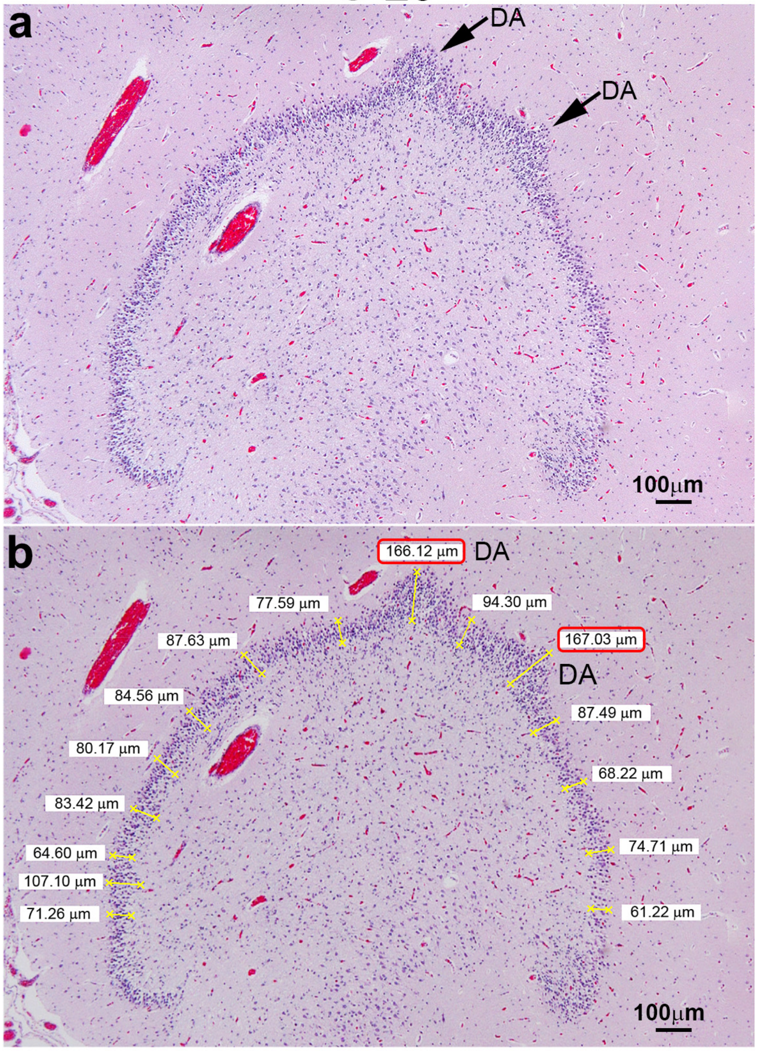

Mean Width including DA = 91.7+/-31.5 µm
Mean Width excluding DA = 80.17+/-12.7 µm

Supplementary Fig. 6
